# Extensive Tracking of Nomadic Waterbird Movements Reveals an Inland Flyway

**DOI:** 10.1002/ece3.70668

**Published:** 2024-12-06

**Authors:** Heather M. McGinness, Micha V. Jackson, Luke Lloyd‐Jones, Freya Robinson, Art Langston, Louis G. O'Neill, Shoshana Rapley, Melissa Piper, Micah Davies, Jessica Hodgson, John M. Martin, Richard Kingsford, Kate Brandis, Veronica Doerr, Ralph Mac Nally

**Affiliations:** ^1^ CSIRO Environment Canberra Australian Capital Territory Australia; ^2^ CSIRO Data61 Brisbane Queensland Australia; ^3^ CSIRO Health and Biosecurity Canberra Australian Capital Territory Australia; ^4^ Fenner School of Environment and Society The Australian National University Canberra Australian Capital Territory Australia; ^5^ CSIRO Agriculture Canberra Australian Capital Territory Australia; ^6^ Royal Botanic Gardens Sydney New South Wales Australia; ^7^ University of New South Wales Sydney New South Wales Australia; ^8^ University of Canberra Canberra Australian Capital Territory Australia; ^9^ School of Biosciences The University of Melbourne Parkville Victoria Australia

**Keywords:** environmental water, movement ecology, Murray–Darling basin, royal spoonbill, satellite telemetry, straw‐necked ibis

## Abstract

Waterbirds are highly mobile and have the ability to respond to environmental conditions opportunistically at multiple scales. Mobility is particularly crucial for aggregate‐nesting species dependent on breeding habitat in arid and semi‐arid wetlands, which can be ephemeral and unpredictable. We aimed to address knowledge gaps about movement routes for aggregate‐nesting nomadic waterbird species by tracking them in numbers sufficient to make robust assessment of their movement patterns. We hypothesised that analysis of long‐distance movements would identify common routes with consistent environmental features that would be useful as context for conservation management. We used GPS satellite telemetry to track the movements of 73 straw‐necked ibis ( *Threskiornis spinicollis* ) and 42 royal spoonbills ( *Platalea regia* ) over 7 years (2016‐2023). We used these data to identify long‐distance movements and to demarcate and characterise movement routes. We identified common routes used by both species, including a ‘flyway’ over 2000 km long, spanning Australia's Murray–Darling Basin from the south‐west to the north‐east. This flyway connects important breeding sites and is characterised by flat, open/unforested areas with low elevations of < 350 m and mid to high rainfall. The flyway corresponds to an area west of Australia's Great Dividing Range, which appears to act as a low‐permeability barrier to the movement of both species. Identification of an inland flyway for waterbirds in Australia provides important context for multi‐jurisdictional cooperation and strategic management. Where resources are limited, water and wetland management efforts (e.g., environmental watering) should be preferentially located within this route. Similarly, targeting threat mitigation within common movement routes may have disproportionate importance for long‐term population viability. Given the widespread distribution of similar species globally, there are likely to be other flyways worthy of scientific and conservation management attention that could be identified using our approach.

## Graphical abstract

We used GPS satellite telemetry to track the movements of 73 straw‐necked ibis ( *Threskiornis spinicollis* ) and 42 royal spoonbills ( *Platalea regia* ) over 7 years. We identified common routes used by both species, including an ‘inland flyway’ spanning Australia's Murray–Darling Basin from the south‐west to the north‐east. This flyway connects important breeding sites and is characterised by flat, open/unforested areas with low elevations of < 350 m and mid to high rainfall. The flyway also aligns with the area west of Australia's Great Dividing Range, which appears to act as a low‐permeability barrier to the movement of both species.

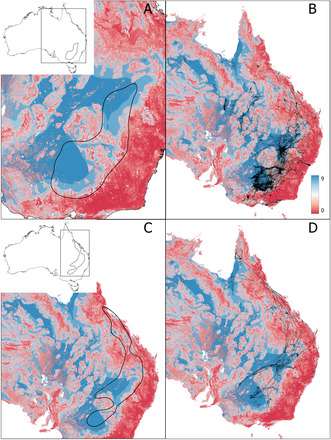

## 1 Introduction

Inland wetlands support a disproportionately large amount of global biodiversity (Dudgeon et al. 2006) and provide enormously valuable ecosystem services (Davidson et al. 2019). However, many are poorly protected, have been drastically altered by human activity, or are facing ongoing interacting threats including overexploitation, pollution, flow modification, habitat destruction and degradation and invasive species (Dudgeon et al. 2006; Kingsford, Bino, and Porter 2017; Reis et al. 2017). Inland wetlands are particularly important in arid and semi‐arid areas for aggregate‐nesting waterbirds^1^ such as Ciconiiformes (egrets, herons, ibis and spoonbills) and Pelecaniformes (cormorants and pelicans) that depend on large areas being flooded for breeding and for sufficient food resources; consequently, these species can be highly sensitive to changes in flood regimes inland (Arthur et al. 2012; Carrick 1962; Francis, Kingsford, and Brandis 2022; Kushlan 1993). Conservation management of such species often focuses on ameliorating the effects of flood regime change to ensure that sufficient habitat is available to support population processes, both short‐term and long‐term.

Australia is the world's driest inhabited continent and is generally water‐poor, with natural wetlands covering only c. 4%–5% of its surface (Bino, Kingsford, and Brandis 2016) and with large, often unpredictable, weather amplitudes causing extreme fluctuations in water availability (Roshier et al. 2001; Williams 2017). Consequently, Australia's aggregate‐nesting waterbirds have adapted by developing opportunistic movement strategies such as nomadism and breeding responses to flooding that can result in spectacularly large numbers of nesting birds at certain sites in wet years, a phenomenon linked to the high productivity of ephemeral wetlands when flooded (McKilligan 1975; Roshier et al. 2001). Many of the largest waterbird breeding aggregations on the Australian continent are located within the Murray–Darling Basin (MDB), which, like many inland wetland systems globally, has experienced significant human pressure. Natural ecosystems within the MDB have experienced significant environmental damage from overallocation of water for consumptive uses over a long period (Hart et al. 2021). Numerous ecological indicators reflect long‐term ecological deterioration of the MDB, exacerbated by droughts such as the Millennium Drought 1997–2010 (van Dijk et al. 2013), which are predicted to worsen with climate change (Brookes et al. 2023; Kingsford, Bino, and Porter 2017). Significant declines in waterbird abundance, breeding frequency, and breeding success have been documented in the MDB (Brandis et al. 2018; Kingsford, Bino, and Porter 2017; Kingsford and Thomas 1995). As a result, the MBD and its inland aggregate‐breeding waterbirds and their habitats are the subject of significant management focus (Connell and Grafton 2011; Hart et al. 2021), including the provision of environmental water (Arthington et al. 2018, 2006; Swirepik et al. 2016), which is water allocated and managed specifically to improve the health of rivers, wetlands and floodplains and their ecological communities.

The effective placement and timing of water management resources for waterbirds requires a detailed understanding of where and when waterbirds require these resources. While major nesting sites of aggregate‐nesting breeding waterbirds are relatively well mapped (Kingsford and Porter 2009; McGinness, Brooks, and Hale 2023; McKilligan 1975), waterbird movements outside the breeding season and the degree of connectivity among breeding sites are relatively poorly understood, particularly in terms of long‐distance movement patterns (Kingsford and Norman 2002; McGinness et al. 2024a). Many aggregate‐nesting waterbirds travel hundreds to thousands of km between breeding events, crossing multiple jurisdictions (Carrick 1962; McKilligan 1975; Nicol, Lloyd‐Jones, and McGinness 2024; Roshier, Asmus, and Klaassen 2008). For such highly mobile species, an understanding of movement routes is essential for the identification of critical habitats for connectivity, prioritisation of management actions and jurisdictional planning and cooperation (McGinness et al. 2024a). Advances in GPS telemetry technology now enable high spatio‐temporal resolution data collection for long periods (months or years), which can overcome limitations in traditional movement data methods, such as banding studies. This is highly relevant for aggregate‐nesting waterbirds in Australia, for which resight data are rare, with < 0.8% of ibis and spoonbills banded seen again after dispersal (ABBBS 2020).

In this study, we aimed to address major knowledge gaps about Australian waterbird movement routes by tracking nomadic aggregate‐nesting waterbirds using GPS satellite telemetry in numbers sufficient to make robust assessment of their dispersal patterns and thereby enhance evidence‐based management. Across 7 years, we tracked the movements of 115 individuals of two species frequently targeted for water management in the MDB: straw‐necked ibis (SNI, *Threskiornis spinicollis* ) and royal spoonbill (RSB, *Platalea regia* ). These species are distributed across Australia, with abundances highest in eastern and northern states and territories, and are also present in lower numbers in New Zealand, New Guinea and Indonesia (Marchant and Higgins 1990). We hypothesised that analysis of long‐distance movements would identify common routes with consistent environmental features that would be useful as context for conservation management.

## 2 Methods

We tracked 115 individuals (73 SNI and 42 RSB) from 2016 to 2023, comprising 41,110 days (cumulative among individuals) of tracking data. We performed all analyses using R Statistical Software version 4.4.0 (R Core Team 2024).

### 2.1 Movement Tracking

**FIGURE 1:**
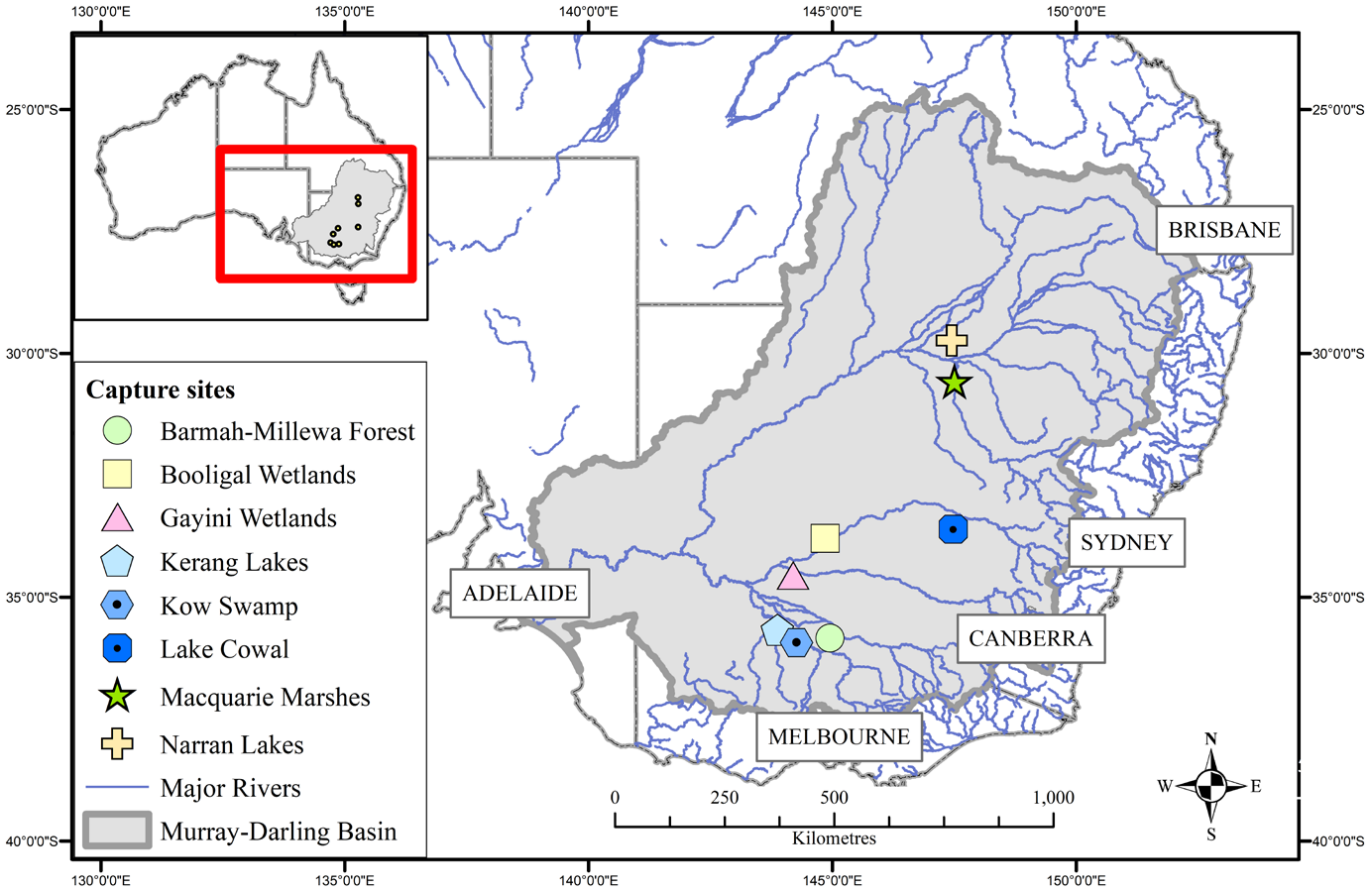
Location of capture sites (2016–2023) of 115 straw‐necked ibis and royal spoonbill caught during aggregate‐breeding events and tracked for > 30 days following nest site dispersal. Inset map shows the location of the MDB and capture sites within Australia.

We deployed transmitters on SNI and RSB at eight breeding sites between 2016 and 2023 (Figure 1, Table S1). All research protocols were approved by an authorised Animal Care and Ethics Committee, according to the Australian code of practice for the care and use of animals for scientific purposes. On‐ground fieldwork activities were conducted under New South Wales and Victoria Scientific Licences 102180 and 10010534. We captured birds either by hand, with leg‐nooses, or by using a net launcher. We attached transmitters as a ‘backpack’ using harnesses made of Teflon ribbon or Spectra ribbon (Bally Ribbon Mills), fitted either as wing‐loops with a join at the keel (SNI and some RSB) or as leg‐loops (RSB). Harness design was based on designs used for other species (Jirinec, Rodrigues, and Amaral 2021; Karl and Clout 1986; Roshier and Asmus 2009; Thaxter et al. 2014) modified and improved over time. Transmitters weighed 12–40 g, ranging from < 1% to 5% of bird bodyweight. We used solar‐powered GPS transmitters with a fix resolution of 15–26 m and fix frequency ranging from 1 minute to 6 h (depending on transmitter type and programmed duty cycle). Data were transmitted via either the Argos satellite network (for Geotrak units) or the 3G network (for Ornitela and Druid units). We considered the duty cycle in analyses, with interpolation or down‐scaling applied when appropriate.

### 2.2 Data Pre‐Processing

To ensure that results reflected longer‐term non‐breeding movements, we limited our analysis to birds for which > 30 days of data were available after the date at which they dispersed from the breeding site where they were captured. Data for nesting adults and for adults and juveniles still within breeding sites after capture but before dispersal were removed from the dataset.

### 2.3 Identifying Long‐Distance Movements

Long‐distance movements were considered to be mutually exclusive with periods of residency, which we defined as short‐distance foraging trips (< 10 km) from a relatively static locale. We extracted long‐distance movements from the dataset separately for each species to account for their ecology. Since SNI roost overnight, we created lines based on each bird's midnight to midnight telemetry points to reflect their long‐distance movements. Since RSB forage and travel at night, we created lines based on the midday‐to‐midday points telemetry points to reflect their long‐distance movements.

Next, we used Hidden Markov Modelling (HMM) to identify movement classes from the prepared tracking data with the goal of separating long‐distance flight movements from foraging/roosting movements. We focussed on the method of Patterson et al. (2009) implemented in the *moveHMM* R package (Michelot et al. 2016) in R. The primary assumptions for using movement data in HMMs are negligible measurement error in positions and regular sampling units (e.g., one positional observation per hour). Post‐quality control, we considered the measurement error negligible. Non‐measured 24 h positions were imputed with missing values. The ‘prepData’ function was used to calculate step distances and angles between points.

The *moveHMM* package requires initial parameters for the step length (gamma distribution) and turning angle (von Mises distribution) distributions. We investigated whether two, three, four, five or six states best modelled the data. For each bird, six runs of the ‘fitHMM’ function were conducted using random starting parameters, ensuring exploration of reasonable starting values. To choose the number of states to make inferences from the HMM for each species, we computed the AIC using the base R *stats* package and compared models through both the AIC value and consistency and visual inspections of the state predictions for each bird as classified lines per track and visual fits of step length and turning angle distributions. We also compared the separation of long distance movements from the HMM method with results from a simple thresholding approach to divide the tracking data, with long‐distance movements defined as the median movement length among all lines plus two standard deviations.

### 2.4 Identification of Common Routes

We used the ‘kernelUD’ function from the R package *adehabitatHR* (Calenge 2022) to estimate the utilisation distribution for all individuals of each species using the kernel density estimation method (KED; Worton 1989). We applied the KED method to tracks identified as long‐distance movements through the best‐fit HMM and the thresholding method. We used a global *h‐*value smoothing parameter (*href*) estimated by the ad hoc method to generate the KEDs (Schuler et al. 2014). For each species, we generated 95%, 90%, 80%, 70%, 60% and 50% KED contours. We plotted the KED contours for each species in base R (Figure S2.1). After reviewing the KEDs, we selected the 50% contour (KED50) as the core utilisation area to define the common movement route for each species (Fleming, Calabrese, and Dray 2016). We quantified the representativeness of the KED50 for individuals and species by calculating:

- For individuals: the number of GPS location fixes from the individual that fell within the KED50/total number of GPS location fixes for the individual.
- For species: the number of GPS location fixes from all individuals of the species that fell within the KED50/total number of GPS location fixes from all individuals of the species.

We also undertook a sensitivity analysis of the *href* value to see how volatile the KED50s were if different *href* smoothing parameter estimates were used. For each species, we generated a KED50 using:

- 70% of the minimum *href* value among all individuals of the species (Jourdan et al. 2021);
- mean and median *href* values for all individuals of the species; and
- 30%, 50% and 70% of the global *href* value estimated for all individuals (Figure S2.2).

We also calculated the proportion of points sampled from the dataset of long‐distance movement lines identified through the HMMs that lie within KED50s generated using the different *href* values (Table S1). We found that the KED50 generated by using the *href* value 70% of the minimum individual *href* produced unrealistically small KED50s; otherwise, KED50 values were similar across all other values generated (i.e., insensitive; Table S1, Figure S2.2), so we proceeded with using the ad hoc smoothing parameter for a global *href* value (i.e., one calculated for all individuals of each species) to generate the KED50s for further analysis.

### 2.5 Characterising Habitat and Climatic Features of Movement Routes

For each species, we sampled the intersection of the KED50 with known important aggregate‐breeding waterbird nesting sites (McGinness, Brooks, and Hale 2023). We then assessed the KED50 for each species against environmental covariates to identify habitat preferences during long‐distance movements. To do so, we tabulated values from: (i) inside the KED50 for each species and (ii) inside a bounding box created around the tracks of each species to demarcate the entire movement range of the species, for the following datasets:

- Multiresolution Valley Bottom Flatness (MrVBF), a topographic index that identifies areas of deposited material at multiple scales on the basis that valley bottoms occur at low elevations and are flat relative to their surroundings with larger valley bottoms flatter than smaller valley bottoms (Gallant, Dowling, and Austin 2016; Gallant and Dowling 2003); index values presented in Table S1.
- National elevation values (continuous dataset of elevation measured in metres above the mean sea level rounded to the nearest metre) sourced from The Multi‐Criteria Analysis Shell for Spatial Decision Support (Howorth 2022) binned into percentiles based on the bounding box values.
- Monthly rainfall averaged over 2011–2020 and binned into deciles over the whole of Australia (Australian Gridded Climate Data; Evans et al. 2020).

### 2.6 Movement Routes in Wet Versus Dry Years

Given the importance of water flow and inundation to the life cycles of SNI and RSB, we compared the characteristics of tracks and KED50s in wet and dry years. To classify years as wet or dry, we examined the extent of drought in the MDB from annual rainfall deficiency (compared to the long‐term average, since 1900) map products from the Australian Government Bureau of Meteorology (Figure S2.3). Based on these maps, we classified 2017–2018, 2018–2019 and 2019–2020 as dry years (substantial areas of the MDB had rainfall deficits) and 2016–2017, 2020–2021, 2021–2022 and 2022–2023 as wet years (little of the MBD had rainfall deficits; Figure S2.3).

## 3 Results

### 3.1 Identifying Long‐Distance Movements and Demarcating Common Routes

For SNI, HMMs using 2–6 states for identifying long‐distance movements produced very similar long distance movement classifications in terms of mean and variance for the longest movement class in each model (Table S1). Furthermore, subsequent KDE50s from each of the models were extremely similar (Figure S2.4). Therefore, we focussed on the results from the five‐state model, which had the lowest AIC and generated four smaller movement classes with a mean distance of < 3 km and a movement class with a mean distance of ~48 ± 71 km, which was close to the overall mean plus one standard deviation computed from the raw data (Table S1). The thresholding approach yielded a dataset of long‐distance movements > 87 km (Table S1). For RSB, results from the 2–6 state models were also very similar in terms of largest movement class and subsequent KDE50 estimation (Table S1, Figure S2.4). We focussed on the results from the six‐state model (lowest AIC), which generated five smaller movement classes with a mean distance of < 4 km and a movement class with a mean distance of ~43 ± 73 km (Table S1). The thresholding approach yielded a dataset of long‐distance movements > 79 km (Table S1). The concordance between classes generated by the lowest AIC fit HMM gave us strong confidence that the movement class with the largest mean distance represented long‐distance movements. The KED50s generated from the lowest AIC HMM and thresholding datasets were very similar to those generated using the threshold approach (Figure S2.5). Therefore, subsequent analysis was only conducted using the KEDs generated from the HMM.

**FIGURE 2:**
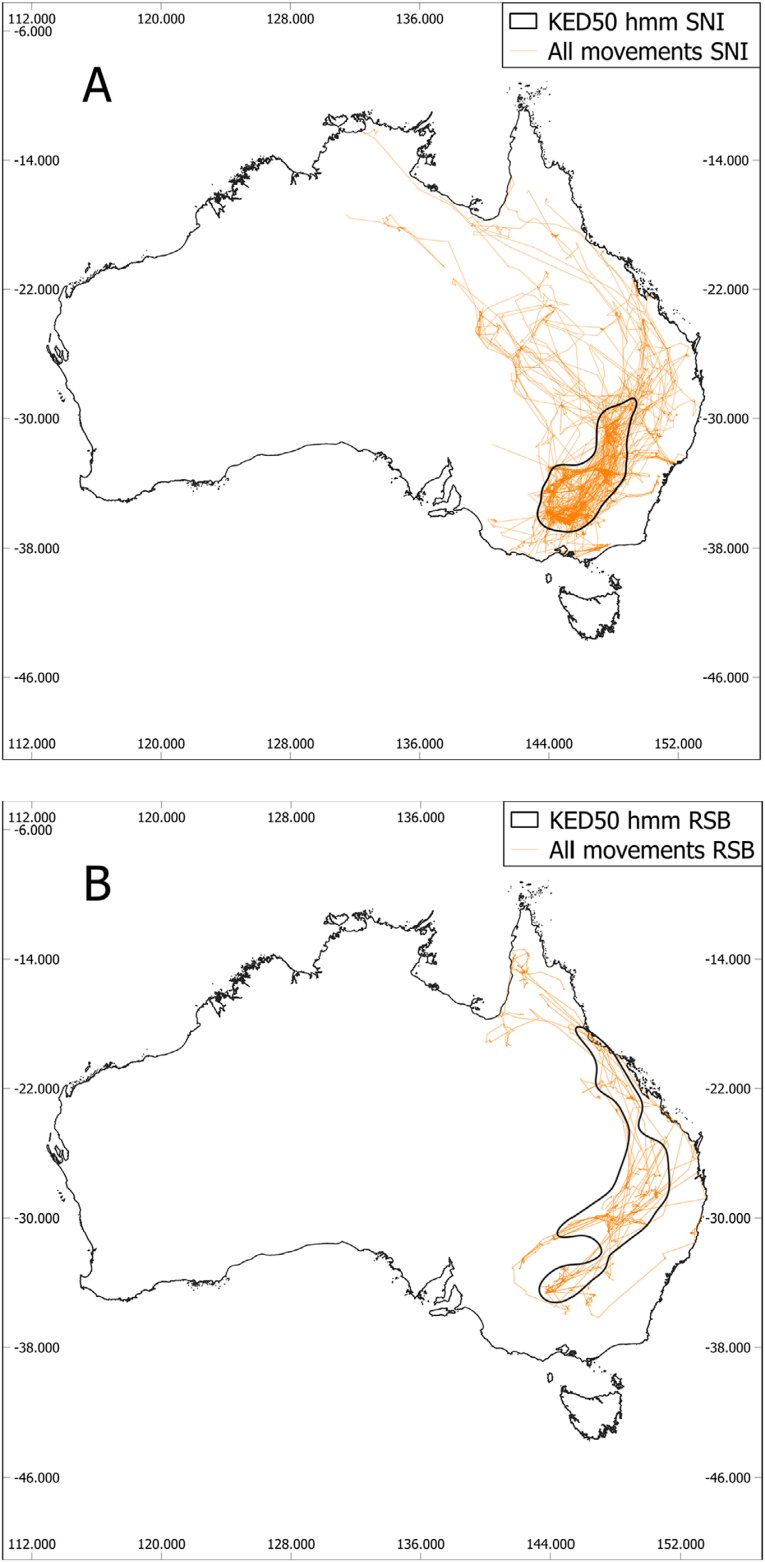
Satellite map of Australia with the 50% contour of the kernel density estimate (KED50) derived from the fourth movement class from a five‐state Hidden Markov Model (straw‐necked ibis; A) and sixth movement class from a six‐state Hidden Markov Model (royal spoonbill; B) with all unclassified movements shown (orange).

Of GPS location fixes from individuals, 61.7% ± 37.3% (SNI) and 47.8% ± 43.3% (RSB) were within the KED50. At the species level, 50.7% (SNI) and 55.0% (RSB) of GPS location fixes from all individuals combined occurred within the KED50 (Figure 2). The KED50 for SNI was more concentrated in south‐eastern Australia with most movements occurring in central NSW and north‐central Victoria (Figure 2A). The KED50 for RSB was more elongated and extended spanning central NSW through inland areas of south‐eastern Queensland to coastal areas of north‐east Queensland (Figure 2B). When overlayed, about 50% of the SNI KED50 overlapped with the RSB KED50 and about 28% of the RSB KED50 overlapped with the SNI KED50.

### 3.2 Characterising Movement Routes

**FIGURE 3:**
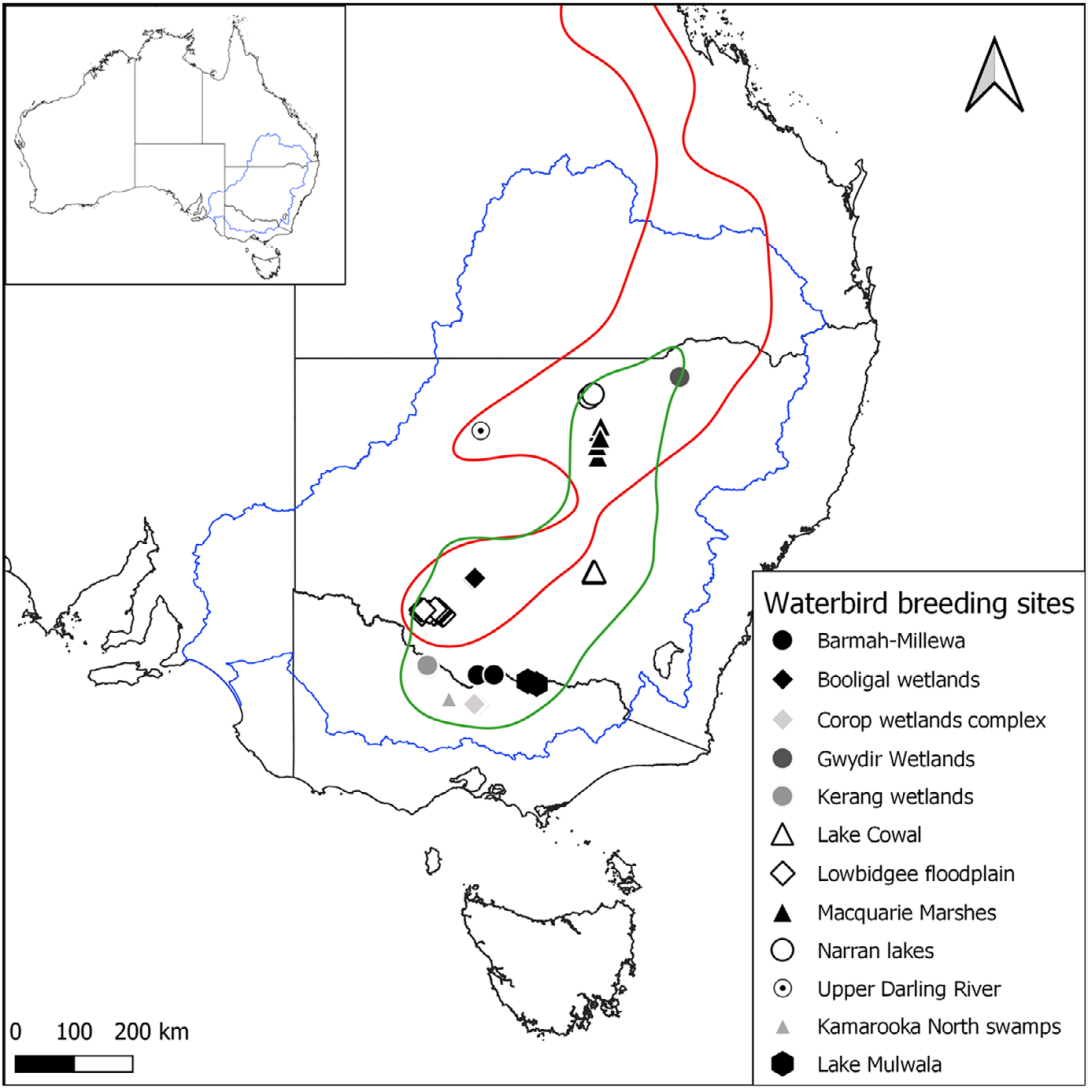
Map of the KED50 for straw‐necked ibis (green) and royal spoonbill (red) showing the locations of important breeding sites for these species (identified in McGinness, Brooks, and Hale 2023) and the boundary of the MDB (blue). Australian state boundaries demarcated in black.

Long‐distance movements by both species as represented by the KED50 areas predominantly occurred in north‐easterly or south‐westerly directions. For both species, the KED50 shows a common movement route that connects several important aggregate‐nesting waterbird breeding sites, including (from north to south): the Gwydir Wetlands, Narran Lakes, the Macquarie Marshes, Lake Cowal, Booligal Wetlands and the Lowbidgee Floodplain (Figure 3; breeding site locations from McGinness, Brooks, and Hale 2023). For SNI, it also connects the Kerang Lakes, Barmah‐Millewa Forest, Lake Mulwala, Kamarooka North Swamps and the Corop Wetlands Complex (Figure 3). For RSB, it also includes Upper Darling River (Figure 3).

**FIGURE 4:**
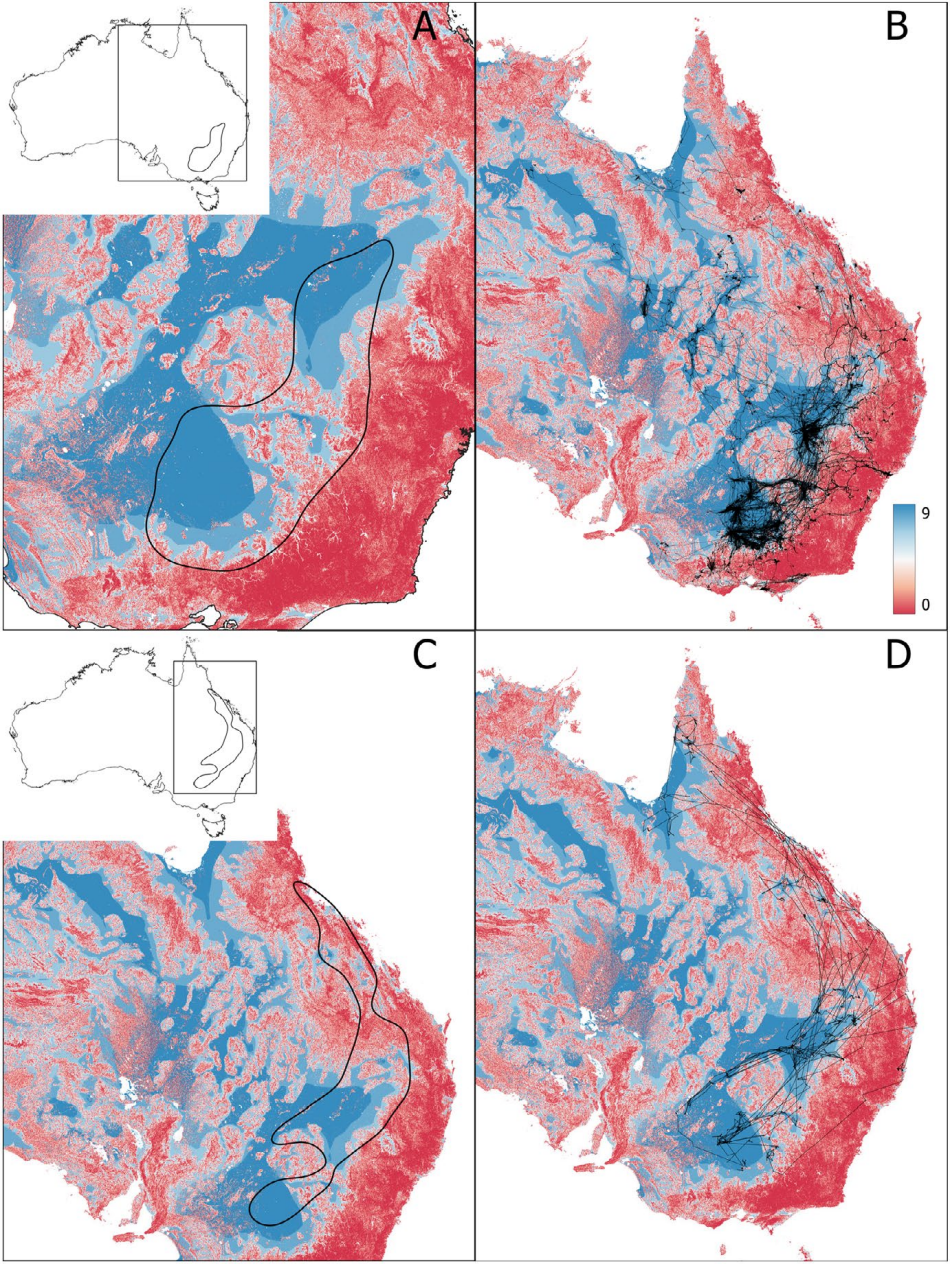
Map of Australia showing the Multiresolution Valley Bottom Flatness index (MrVBF) and (i) the KED50 for straw‐necked ibis (A); (ii) all long‐distance movements of straw‐necked ibis (B); (iii) the KED50 for royal spoonbill (C); and (iv) all long‐distance movements of royal spoonbill (D). Insets show the KED50 and a bounding box of all movements on a map of Australia. MrVBF values are shown on a gradient from lowest (red) to highest (blue); refer to Table S1 for index value interpretation.

**FIGURE 5:**
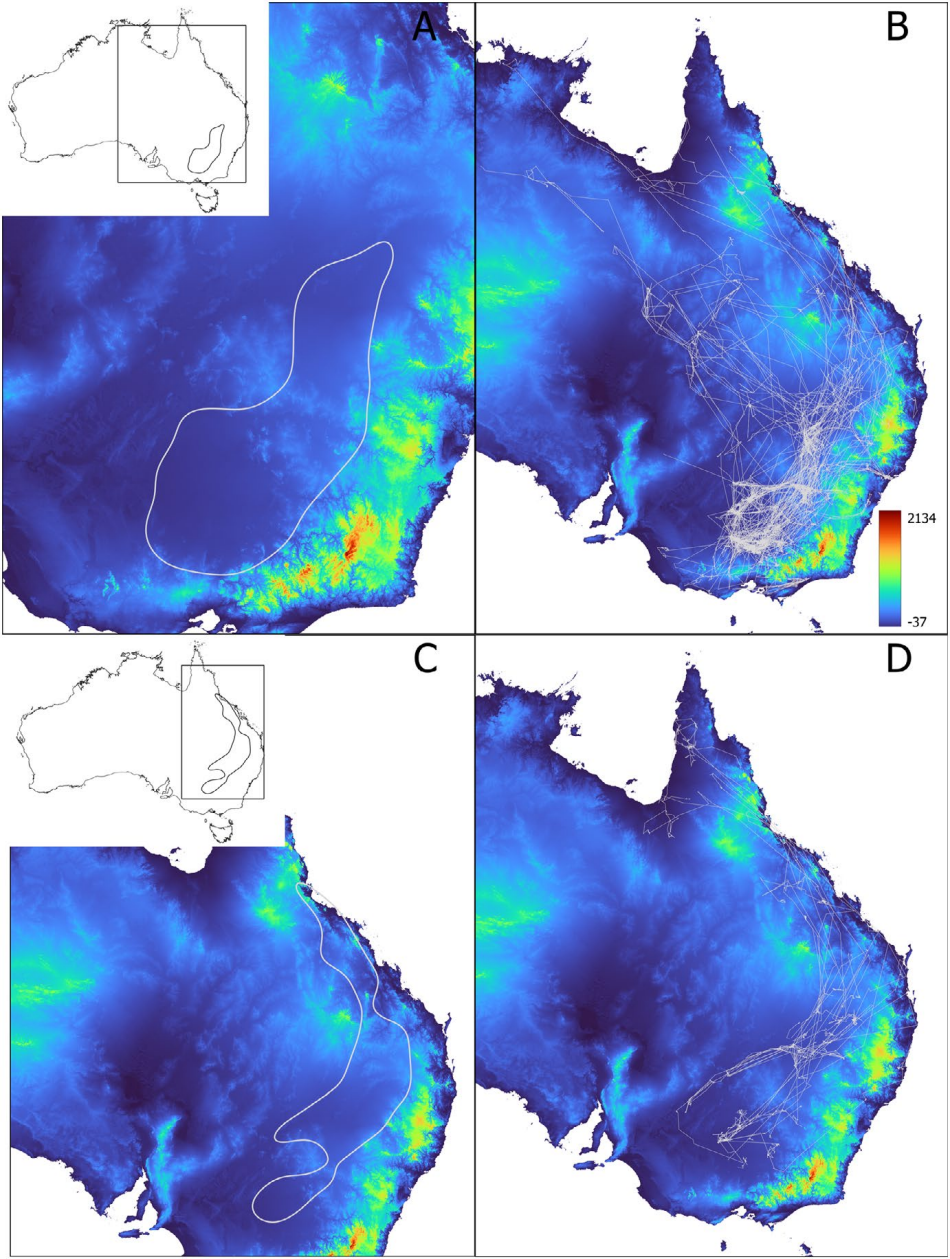
Map of Australia showing elevation and: (i) the KED50 for straw‐necked ibis (A); (ii) all long‐distance movements of straw‐necked ibis (B); (iii) the KED50 for royal spoonbill (C); (iv) all long‐distance movements of royal spoonbill (D). Insets show the KED50 an bounding box of all movements on a map of Australia. Elevation values (metres) are shown on a gradient from lowest (blue) to highest (red).

**FIGURE 6:**
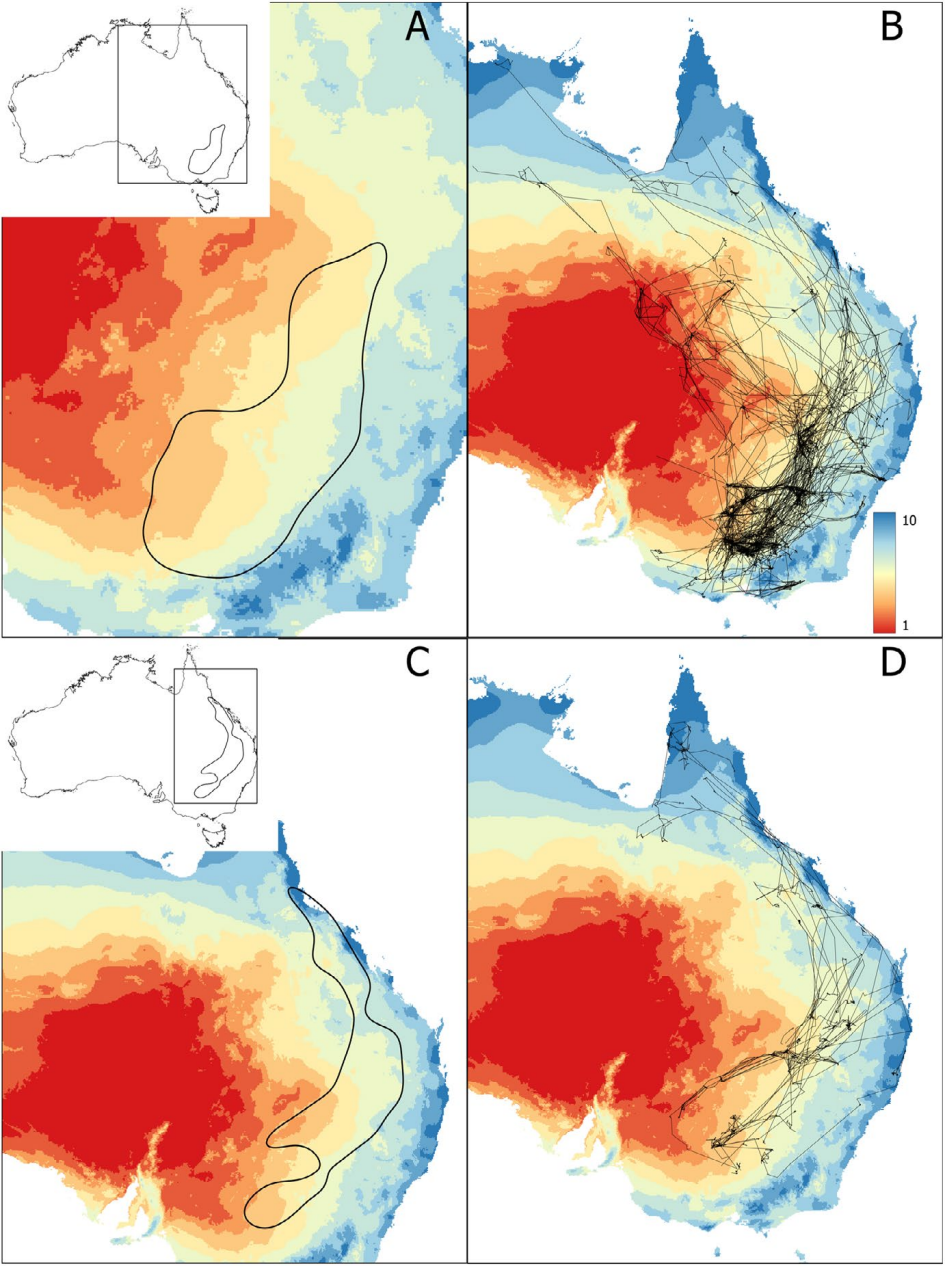
Map of Australia showing annual rainfall (millimetres) values averaged over 2011–2020 and binned into deciles (millimetres) over the whole of Australia and (i) the KED50 for straw‐necked ibis (A); (ii) all long‐distance movements of straw‐necked ibis (B); (iii) the KED50 for royal spoonbill (C); (iv) all long‐distance movements of royal spoonbill (D). Insets show the KED50 an bounding box of all movements on a map of Australia. Rainfall values (millimetres) are shown on a gradient from lowest (red) to highest (blue).

For both species, the distribution of MrVBF values inside the KED50 and the bounding box clearly deviated (chi‐square goodness‐of‐fit test: SNI—stat. = 1.27e+08, df = 9, *p*‐value < 2.2e‐16; RSB—stat. = 5.1e+05, df = 9, *p*‐value < 2.2e‐16). The distribution within the KED50 was overrepresented relative to the background distribution for high MrVBF classes and underrepresented or low MrVBF classes (Table 1, Figure 4). This indicates that birds favoured flat, low‐elevation and open/unforested areas. These results were reinforced by the distribution of elevation values inside the KED50 and bounding box (chi‐square goodness‐of‐fit test: SNI—stat. = 9.39e+06, df = 8, *p*‐value < 2.2e‐16, RSB—stat. = 7.73e+06, df = 8, *p*‐value < 2.2e‐16). The distribution inside the KED50 compared with the distribution inside the bounding box was inflated for elevations between ~85 and 195 m for SNI and between ~155 and 195 m for RSB and deflated above c. 350 m for SNI and above c. 500 m for RSB (Table 2, Figure 5), again suggesting an avoidance of higher elevations but with RSB using more high elevation areas than SNI. For rainfall, the distribution of values inside the KED50 and bounding box also differed (chi‐square goodness‐of‐fit test: SNI—stat. = 1.96e+04, df = 9, *p*‐value < 2.2e‐16, RSB—stat. = 1.95e+04, df = 9, *p*‐value < 2.2e‐16), with the distribution inside the KED50 highly deflated compared with the distribution inside the bounding box for low rainfall values of ~0–300 mm annual rainfall and inflated for rainfall values of ~300–550 mm annual rainfall for both species (Table 3, Figure 6).

**TABLE 1:** Distribution of Multiresolution Valley Bottom Flatness index values inside the KED50 contour and bounding box.

MrVBF value	9	8	7	6	5	4	3	2	1	0
KED50 contour—SNI	0.33	0.21	0.08	0.07	0.05	0.04	0.06	0.04	0.01	0.11
Bounding box—SNI	0.13	0.11	0.10	0.08	0.07	0.06	0.12	0.07	0.02	0.24
KED50 contour—RSB	0.25	0.14	0.07	0.07	0.06	0.05	0.10	0.06	0.01	0.19
Bounding box—RSB	0.15	0.11	0.09	0.08	0.06	0.06	0.11	0.07	0.02	0.25

*Note:* The bounding box represents the entire movement range of each species. Coloured cells indicate substantial overrepresentation (green—suggesting selection of that MRVBF zone) and underrepresentation (red—suggesting avoidance of that MRVBF zone) relative to the background (i.e., bounding box values). MRVBF index values are presented in Table S1.

Abbreviations: RSB = royal spoonbill; SNI = straw‐necked ibis.

**TABLE 2:** Distribution of elevation (metres above the sea level) values (binned into percentiles based on the bounding box values) inside the KED50 contour and bounding box representing the entire movement range of each species.

Elevation	46–84 m	85–119 m	120–155 m	156–194 m	195–232 m	233–274 m	275–348 m	349–497 m	> 497 m
KED50 contour—SNI	0.10	0.22	0.22	0.15	0.12	0.09	0.07	0.03	0.01
Bounding box—SNI	0.11	0.11	0.11	0.11	0.11	0.11	0.11	0.11	0.11
KED50 contour—RSB	0.06	0.10	0.14	0.16	0.14	0.13	0.15	0.10	0.02
Bounding box—RSB	0.11	0.11	0.11	0.11	0.11	0.11	0.11	0.11	0.11

*Note:* Coloured cells indicate substantial overrepresentation (green) and underrepresentation (red) relative to the background (i.e., bounding box values).

Abbreviations: RSB = royal spoonbill; SNI = straw‐necked ibis.

**TABLE 3:** Distribution of annual rainfall (millimetres) values (averaged over 2011–2020 and binned into deciles over the whole of Australia) inside the KED50 contour and bounding box representing the entire movement range of each species.

Rainfall	0–210 mm	211–264 mm	265–297 mm	298–352 mm	353–436 mm	437–552 mm	552–736 mm	737–979 mm	980–1317 mm
KED50 contour—SNI	0.00	0.00	0.00	0.20	0.36	0.34	0.09	0.01	0.00
Bounding box—SNI	0.26	0.07	0.06	0.07	0.09	0.11	0.10	0.09	0.08
KED50 contour—RSB	0.00	0.00	0.03	0.17	0.20	0.36	0.16	0.03	0.02
Bounding box—RSB	0.19	0.07	0.06	0.08	0.09	0.12	0.12	0.07	0.09

*Note:* Coloured cells indicate overrepresentation (green) and underrepresentation (red) relative to the background (i.e., bounding box values).

Abbreviations: RSB = royal spoonbill; SNI = straw‐necked ibis.

### 3.3 Movement Routes in Wet and Dry Years

For both SNI and RSB, the majority of the long‐distance movements were in wet years. For SNI, 3951 of 4749 (83.2%) long‐distance lines were in wet years, and for RSB, 776 of 811 (95.7%) were in wet years. Consequently, the overall KED50 and the KED50 for wet years were very similar (Figures S2.6 and S2.7).

## 4 Discussion

This is the first time that satellite tracking of aggregate‐nesting waterbird species in Australia has been used to identify common movement routes. Notably, we identified an inland ‘flyway’ used over multiple years by many individuals of two species. Long‐term high‐resolution tracking such as this is critical for understanding bird movement routes in inland regions, particularly in arid and semi‐arid areas and when species movement strategies are dominated by nomadism, as they are here. The remote regions used and relative lack of site fidelity in these species make other techniques, such as leg‐banding and marking, unfeasible and unlikely to be useful, while satellite tracking is the best current way to elucidate movement patterns.

The common route or flyway identified here has a number of defining physical characteristics, including flat, open/unforested areas with low elevations of < 350 m (as represented by MRVBF indices and elevation), and rainfall patterns characteristic of predominantly transitional or semi‐arid climatic zones. These characteristics are associated with a combination of known preferred foraging and roosting habitats, food availability and wind conditions for these species, as described by previous work using this dataset (McGinness, Langston, and Brooks 2020; McGinness et al. 2024b, 2024a; Nicol, Lloyd‐Jones, and McGinness 2024). Both RSB and SNI typically forage in open, shallow, low‐elevation wetlands and floodplains (Marchant and Higgins 1990) and frequently use ephemeral or temporary wetlands and floodplains occurring in semi‐arid zones (McGinness, Langston, and Brooks 2020; McGinness et al. 2024b, 2024a; Nicol, Lloyd‐Jones, and McGinness 2024), which are often more productive and biodiverse than permanently inundated wetlands (Calhoun et al. 2017).

The flyway is bound on the east by Australia's Great Dividing Range (GDR), which is not a definitively mapped continental feature, but is generally represented by a > 300 m elevation contour that runs north–south along the east of the continent (Figure S2.8). The GDR may present a barrier to the movement of ibis and spoonbills from inland parts of the MDB to the wetter south‐eastern coast of New South Wales and north‐eastern coast of Victoria (Figure S2.8). This is consistent with the results of Guay et al. (2012), who investigated waterbird movement across the southern section of the GDR by five other avian families using Australian Bird and Bat Banding Scheme data: Anatidae (ducks, geese and swans), Anhingidae (Darter), Phalacrocoracidae (Cormorants), Ardeidae (egrets, herons and bitterns) and Rallidae (hens, coots, crakes and rails). They found that of the 33 species of these families that were banded inland of the GDR, only 12 species had band‐recovery records on the other side of the range in Victoria, and most of these were ducks, cormorants and cattle egrets ( *Bubulcus ibis* ). Similarly, recorded displacements of ibis and spoonbills from inland across the southern parts of the GDR have been rare, but these species cross the GDR in the north, where the GDR elevation is lower and more broken by valleys. Notably, the RSB movement route extends further north than that of SNI through a relatively lower elevation section of the GDR, at which point the RSB movement route shifts to the coastal side of the GDR (Figure S2.8). For this species, in the northern part of its movement route, the GDR may present a barrier to the movement of RSB from coastal areas of north‐east Queensland to more inland parts of northern Queensland. It is not yet known to what extent SNI and RSBs in more southerly coastal areas east of the GDR are physically or genetically connected to inland populations. If connectivity is very low, the value of such coastal sites as physical or genetic refuges may also be low for the inland populations, increasing the importance of conservation of inland habitats.

While both SNI and RSB used the flyway identified here, our results suggest species differences in long‐distance movement strategies, distances and routes. Both adult and juvenile SNI are highly nomadic and may move in any direction at any time of year (McGinness et al. 2024a); however, many adult and juvenile RSBs move north toward warmer zones in autumn/winter and then remain there for relatively long periods without the degree of movement seen in SNI (Marchant and Higgins 1990; McGinness, Langston, and Brooks 2020; McGinness et al. 2024b, 2024a; Nicol, Lloyd‐Jones, and McGinness 2024). It is likely that most juvenile RSBs stay in northern parts of Australia until reaching breeding age (approximately 3 years old), at which point they are expected to return south to breeding sites. This delayed return by juveniles is generally what occurs in the RSB population in New Zealand (Schweigman 1999; Schweigman, Cash, and Thompson 2014) as well as populations of related spoonbill species elsewhere (e.g., Eurasian spoonbill, references). However, this return has not yet been tracked in Australia, and our tracking has already shown that there are exceptions (as there are in other countries), with some juveniles and adults remaining in the south and behaving nomadically over successive years (McGinness et al. 2024b). There are also species differences in foraging habitats and diet that likely influence their route choices, movement distances and habitat selection (Marchant and Higgins 1990; McGinness et al. 2024a).

Our ability to explore differences in movements in wet and dry years was limited because most of the long‐distance movements in our tracking study were from wet years, but this would be a fruitful area for future research. There was some evidence that RSB movements in wet years went further north than in dry years while SNI movements were more extensive in dry years (Figures S2.6 and S2.7), but this was influenced by the number of each species tagged at different capture sites in different years.

### 4.1 Implications for Management

A ‘flyway’ is a concept originally developed to identify broad regions within which waterbird species move during large‐scale migrations, typically at an inter‐continental scale. The utility of the concept is to represent the complexities of bird movements in a simple and geographical way that can enhance collaboration and cooperation among the stakeholders and jurisdictions within which these birds travel, leading to improvements in species and habitat conservation (Boere and Stroud 2006). Grouping bird movements into flyways has yielded substantive benefits such as providing a non‐legally binding framework for cooperation among diverse state and non‐state actors (Gallo‐Cajiao et al. 2019) and facilitating direct conservation action leading to improved population trajectories (Marcacci et al. 2022). Flyway‐scale cooperation has been further enhanced by advances in tracking technology that have greatly improved knowledge of movement pathways and important habitats (Chan et al. 2019; Marcacci et al. 2022).

Historically, birds have been categorised as using a flyway if they undertake regular biannual movements following a latitudinal direction, moving between distinct breeding and non‐breeding areas with high fidelity to those areas, and all individuals of the species migrate, usually at inter‐continental scales (BirdLife International 2010). We suggest that an analogous framework to the flyway concept is needed for inland continental‐scale movements by nomadic and partially migratory species that do not fit the criteria traditionally used to demarcate global flyways but that are clearly using common routes to engender management and jurisdictional cooperation. These species cross multiple jurisdictions, within which there are many stakeholders, and they also require management of all important habitats among multiple species to meet ‘whole‐of‐life‐cycle’ needs.

A better understanding of common movement routes for such species could significantly improve management by highlighting critical habitat and management zones and hence stakeholders. In this case, the flyway we have identified could be used to prioritise limited water and wetland management resources. For example, environmental watering is likely to have a greater potential impact on connectivity for a larger number of birds (and potentially species) within these routes. Similarly, threats such as habitat loss, fragmentation, pollution and climate change may have a greater effect when occurring within the inland flyway than in other areas. As such, strategic management actions for such threats within the flyway may have disproportionate benefits to long‐term species population growth and viability.

## 5 Conclusion

We identified common long‐distance movement routes for SNI and RSB in the largest waterbird tracking study of its kind in Australia. Crucially, we identified a flyway running longitudinally across eastern Australia. The flyway is characterised by flat, open landscapes at low elevation in the semi‐arid and transitional arid zones. Additionally, the flyway is bounded on the east by the GDR, which may have consequences for population connectivity across this orographic barrier; furthermore, the GDR appears more permeable to RSB than SNI, at least in the north. Knowledge of these routes provides context for multi‐jurisdictional cooperation for prioritisation and application of management efforts such as environmental watering. An understanding of bird movement routes provides crucial insights into the spatial ecology of species of management interest, guiding targeted and effective conservation actions to ensure the long‐term survival of these species and the ecosystems upon which they depend. Given the widespread distribution of similar species globally, there are likely to be other inland flyways worthy of scientific and conservation management attention that could be identified using our approach.

## Author Contributions

**Heather M. McGinness:** conceptualization (lead), data curation (lead), formal analysis (equal), funding acquisition (lead), investigation (lead), methodology (equal), project administration (lead), resources (lead), supervision (lead), validation (lead), visualization (equal), writing – original draft (lead), writing – review and editing (equal). **Micha V. Jackson:** data curation (supporting), formal analysis (equal), methodology (equal), visualization (equal), writing – original draft (equal), writing – review and editing (equal). **Luke Lloyd‐Jones:** data curation (equal), formal analysis (equal), investigation (equal), methodology (equal), visualization (equal), writing – original draft (equal), writing – review and editing (equal). **Freya Robinson:** conceptualization (supporting), data curation (equal), investigation (equal), methodology (supporting), resources (equal), visualization (supporting), writing – original draft (supporting). **Art Langston:** conceptualization (supporting), data curation (equal), formal analysis (supporting), investigation (supporting), methodology (supporting), visualization (supporting). **Louis G. O'Neill:** data curation (supporting), investigation (supporting), writing – review and editing (supporting). **Shoshana Rapley:** data curation (supporting), investigation (supporting), writing – review and editing (supporting). **Melissa Piper:** data curation (supporting), investigation (supporting). **Micah Davies:** data curation (supporting), investigation (supporting). **Jessica Hodgson:** data curation (supporting), investigation (supporting). **John M. Martin:** investigation (supporting), methodology (supporting). **Richard Kingsford:** funding acquisition (supporting), investigation (supporting). **Kate Brandis:** funding acquisition (supporting), investigation (supporting). **Veronica Doerr:** funding acquisition (supporting), investigation (supporting). **Ralph Mac Nally:** funding acquisition (supporting), investigation (supporting), writing – review and editing (supporting).

## Ethics Statement

All research protocols were approved by an authorised Animal Care and Ethics Committee, according to the Australian code of practice for the care and use of animals for scientific purposes. On‐ground fieldwork activities were conducted under New South Wales and Victoria Scientific Licences 102180 and 10010534.

## Conflicts of Interest

The authors declare no conflicts of interest.

## Supporting information


Data S1.

## Data Availability

Tracking data used in this study are viewable on Movebank https://www.movebank.org/. Data and code are available on reasonable request to the author through the CSIRO Data Access Portal https://data.csiro.au/.

## Endnotes

^1^
The authors note here that much of the published literature refers to the same group of species discussed in this manuscript as ‘colonial‐nesting waterbirds’ and their breeding sites as ‘colonies’. In this context, the words ‘colonial’ and ‘colony’ are meant to have a strictly ecological meaning. However, there is increasing recognition in ecology, conservation, and other scientific disciplines that there are historical legacies of discrimination and oppression of marginalised groups within these disciplines, often associated with human colonial history (Chaudhury and Colla 2020; Salomon et al. 2018). In acknowledgement of this history and to avoid negative associations with human colonial history, we have used the term ‘aggregate‐nesting’ and avoided use of the terms ‘colony’ and ‘colonial’ throughout this manuscript.
