# Supplementary material for: Extensive Tracking of Nomadic Waterbird Movements Reveals an Inland Flyway
Source: Ecol Evol. 2024 Dec 6;14(12):e70668. doi: 10.1002/ece3.70668 (PMC11621969; doi:10.1002/ece3.70668)
Supplement: Supplementary file 1 — Data S1. [file ECE3-14-e70668-s001.docx]

# **Supporting Information**

## Appendix 1 – Supporting Tables

Table S1.1 Transmitter deployment summary. Refer to Figure 1 for capture site locations.

| Capture site | Sp | 2016-2017 | 2017-2018 | 2018-2019 | 2019-2020 | 2020-2021 | 2021-2022 | 2022-2023 | 2023-2024 |
| --- | --- | --- | --- | --- | --- | --- | --- | --- | --- |
| Barmah-Millewa Forest | SNI | 8 |  |  |  |  |  |  | 4 |
| Barmah-Millewa Forest | RSB |  | 6 |  |  | 8 |  |  |  |
| Booligal Wetlands | SNI |  |  |  |  |  |  | 4 |  |
| Booligal Wetlands | RSB |  |  |  |  |  |  | 2 |  |
| Gayini Wetlands | SNI |  |  |  |  | 12 |  | 4 |  |
| Gayini Wetlands | RSB |  |  |  |  | 6 |  | 5 |  |
| Kerang Lakes | SNI |  | 7 |  |  |  |  |  |  |
| Kerang Lakes | RSB |  | 1 |  |  |  |  |  |  |
| Kow Swamp | SNI |  | 10 |  | 6 |  |  |  |  |
| Kow Swamp | RSB |  |  |  | 1 |  |  |  |  |
| Lake Cowal | SNI |  |  |  |  |  | 3 |  |  |
| Lake Cowal | RSB |  |  |  |  |  | 1 |  |  |
| Macquarie Marshes | SNI |  | 5 |  |  |  | 6 | 1 |  |
| Macquarie Marshes | RSB |  |  |  |  |  |  | 7 |  |
| Narran Lakes | SNI |  |  |  |  |  | 3 |  |  |
| Narran Lakes | RSB |  |  |  |  |  | 5 |  |  |

Table S1.2 Proportion of points sampled from the dataset of long-distances movement lines identified through Hidden Markov Modelling (HMM; refer to Table S1.4) that lie within KED50s generated using different href values.

| href method | href value | Proportion |
| --- | --- | --- |
| Straw-necked ibis |  |  |
| href with ad-hoc smoothing parameter | 60573 | 0.55 |
| 70% minimum href amongst all individuals | 1277.257 | 0.39 |
| Median h-ref amongst all individuals | 48220.89 | 0.54 |
| Mean h-ref amongst all individuals | 53948.68 | 0.55 |
| 70% global href (top row) | 42401.1 | 0.53 |
| 50% global href (top row) | 30286.5 | 0.52 |
| 30% global href (top row) | 18171.9 | 0.52 |
| Royal spoonbill |  |  |
| href with ad-hoc smoothing parameter | 60573 | 0.65 |
| 70% minimum href amongst all individuals | 1277.257 | 0.10 |
| Median href amongst all individuals | 48220.89 | 0.60 |
| Mean h-ref amongst all individuals | 53948.68 | 0.61 |
| 70% global href (top row) | 42401.1 | 0.63 |
| 50% global href (top row) | 30286.5 | 0.60 |
| 30% global href (top row) | 18171.9 | 0.59 |

Table S1.3 Index values for the Multi-resolution Valley Bottom Flatness (MrVBF). The method for calculating the MrVBF is described in Gallant and Dowling (2003). Each index value of the MrVBF (column 1) is associated with a slope threshold (column 2) and scale (column 3); for each successive index value the slope threshold halves and the scale roughly triples; the interpretation of index values (column 4) is that at each scale a location is considered erosional if it is high in elevation (ranked above the majority of the surrounding cells) or steep (slope greater than the threshold), and depositional otherwise (Gallant et al., 2016).

| MrVBF index value | Threshold slope (%) | Scale | Interpretation |
| --- | --- | --- | --- |
| 0 | 0.0625 | 30 m | Erosional |
| 1 | 0.125 | 30 m | Small hillside deposit |
| 2 | 0.25 | 30 m | Narrow valley floor |
| 3 | 0.5 | 90 m | Less-narrow valley floor |
| 4 | 1 | 270 m | Valley floor |
| 5 | 2 | 800 m | Extensive valley floor |
| 6 | 4 | 2.4 km | Small depositional basin |
| 7 | 8 | 7.2 km | Depositional basin |
| 8 | 16 | 22 km | Large depositional basin |
| 9 |  | 66 km | Extensive depositional basin |

Table S1.4 Results of Hidden Markov Modelling to automatically identify movement classes (two, three, four, five and six states) from a dataset of lines of the maximum distance that a bird had travelled in the 24 hours from midnight to midnight (SNI) or midday to midday (RSB). Table shows the mean, minimum and maximum AIC for each model (n=6 runs) and the mean ± standard deviation (km) for each movement class from the model for that state with the lowest AIC. Rows in bold identify the HMM for each species with the lowest AIC, which was the model used to generate the KE50. Values in bold italics are those that were used as ‘long-distance’ movements.

| Sp | States (#) | Mean AIC | Min AIC | Max AIC | State 1 | State 2 | State 3 | State 4 | State 5 | State 6 |
| --- | --- | --- | --- | --- | --- | --- | --- | --- | --- | --- |
| SNI | 2 | 201264.5 | 201264 | 201267 | 1.0±1.5 | 32.6±51.4 |  |  |  |  |
| SNI | 3 | 192803.8 | 191143 | 201108 | .04±.04 | 1.8±2.1 | 40.3±60.8 |  |  |  |
| SNI | 4 | 174328.8 | 164859 | 191168 | .04±.03 | 0.5±0.6 | 1.8±2.2 | 36.4±56.7 |  |  |
| **SNI** | **5** | **164939.2** | **161714** | **167490** | **.04±.03** | **0.7±0.9** | **2.1±2.7** | ***47.7±70.8*** | **2.5±2.4** |  |
| SNI | 6 | 165176.2 | 159656 | 170292 | .01±.01 | 0.1±.04 | 0.6±0.8 | 2.3±2.9 | 47.8±70.0 | 2.6±2.9 |
| RSB | 2 | 54271.3 | 54243 | 54277 | 1.4±2.1 | 32.1±48.9 |  |  |  |  |
| RSB | 3 | 52357.7 | 51388 | 54297 | 0.1±0.1 | 2.4±3.0 | 41.6±60.4 |  |  |  |
| RSB | 4 | 50787 | 50118 | 51412 | 0.1±0.1 | 2.3±2.9 | 42.2±61.1 | 3.0±3.3 |  |  |
| RSB | 5 | 49698.5 | 49114 | 50309 | .03±.03 | 0.3±0.3 | 3.4±3.9 | 47.6±67.8 | 3.4±3.5 |  |
| **RSB** | **6** | **48682.8** | **44786** | **49806** | **.01±0** | **.04±.03** | **0.3±0.3** | **3.4±5.9** | **3.0±3.9** | ***43.3±73.2*** |

Table S1.5 Summary of distance statistics for the dataset of lines of the maximum distance that a bird had travelled in the 24 hours from midnight to midnight (SNI) or midday to midday (RSB).

| Species | Mean line distance (km) | Median line distance (km) | Standard Deviation (km) | Maximum line distance (km) | Median + 2SD (km) |
| --- | --- | --- | --- | --- | --- |
| SNI | 16.7 | 5.9 | 40.7 | 662.7 | 87.3 |
| RSB | 13.6 | 5.4 | 38.8 | 856.9 | 79.3 |

## Appendix 2 – Supporting Figures


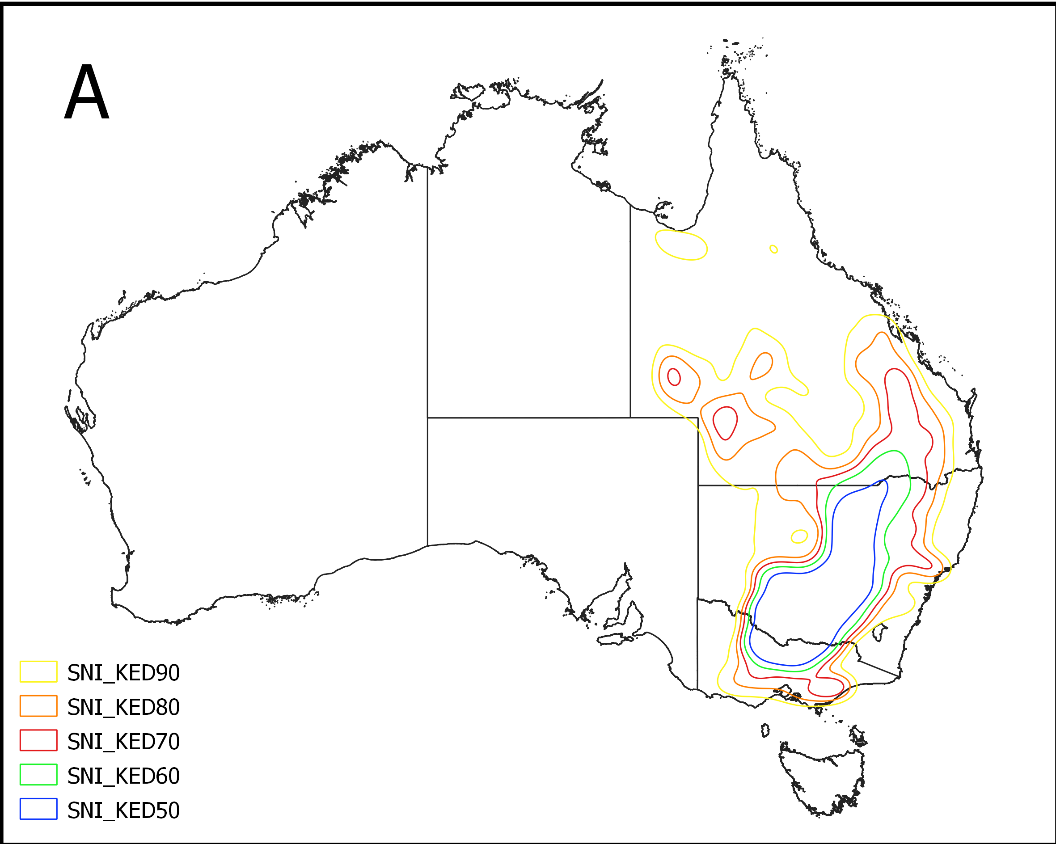


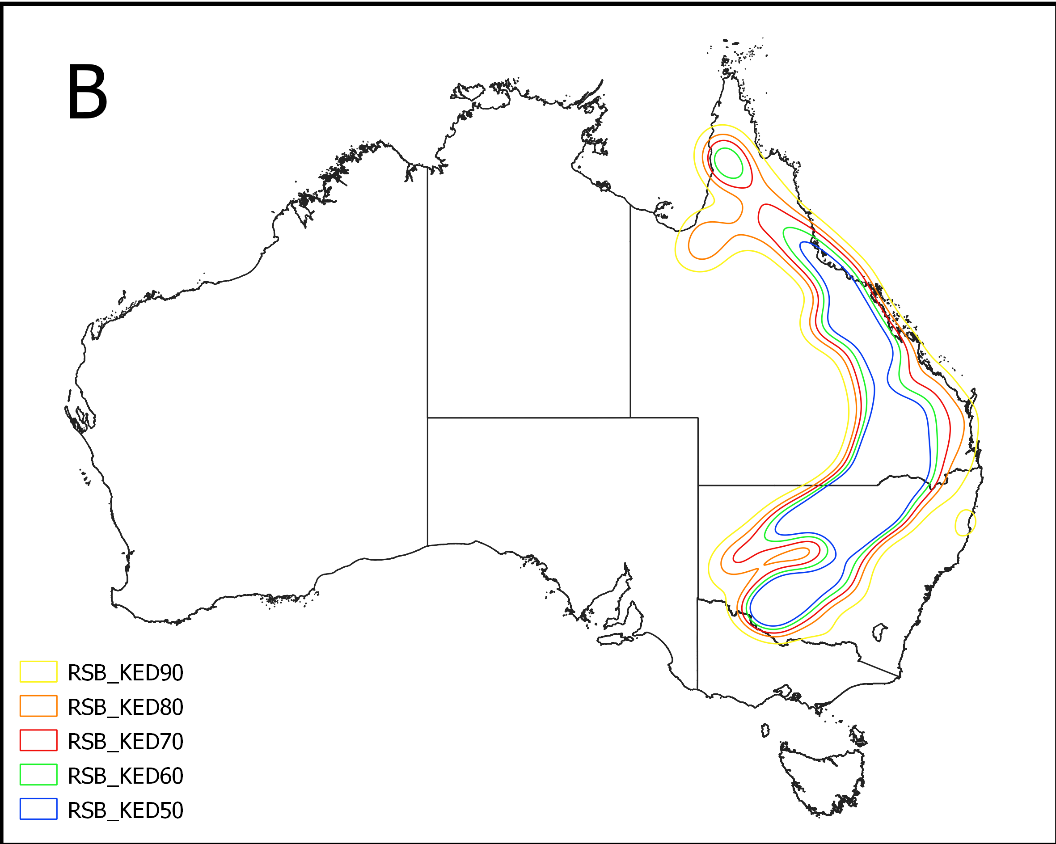


Figure S2.1 Comparison of KED90, KED80, KED70, KED60 and KED50 generated using a dataset of long-distance movements subset from a dataset of lines of the maximum distance that a bird had travelled in the 24 hours from midnight to midnight (SNI) or midday to midday (RSB) based on: i) fourth movement class from a five-state Hidden Markov Model (SNI; A; Table S1.4) and sixth movement class from a six-state Hidden Markov Model (RSB; B; Table S1.4).
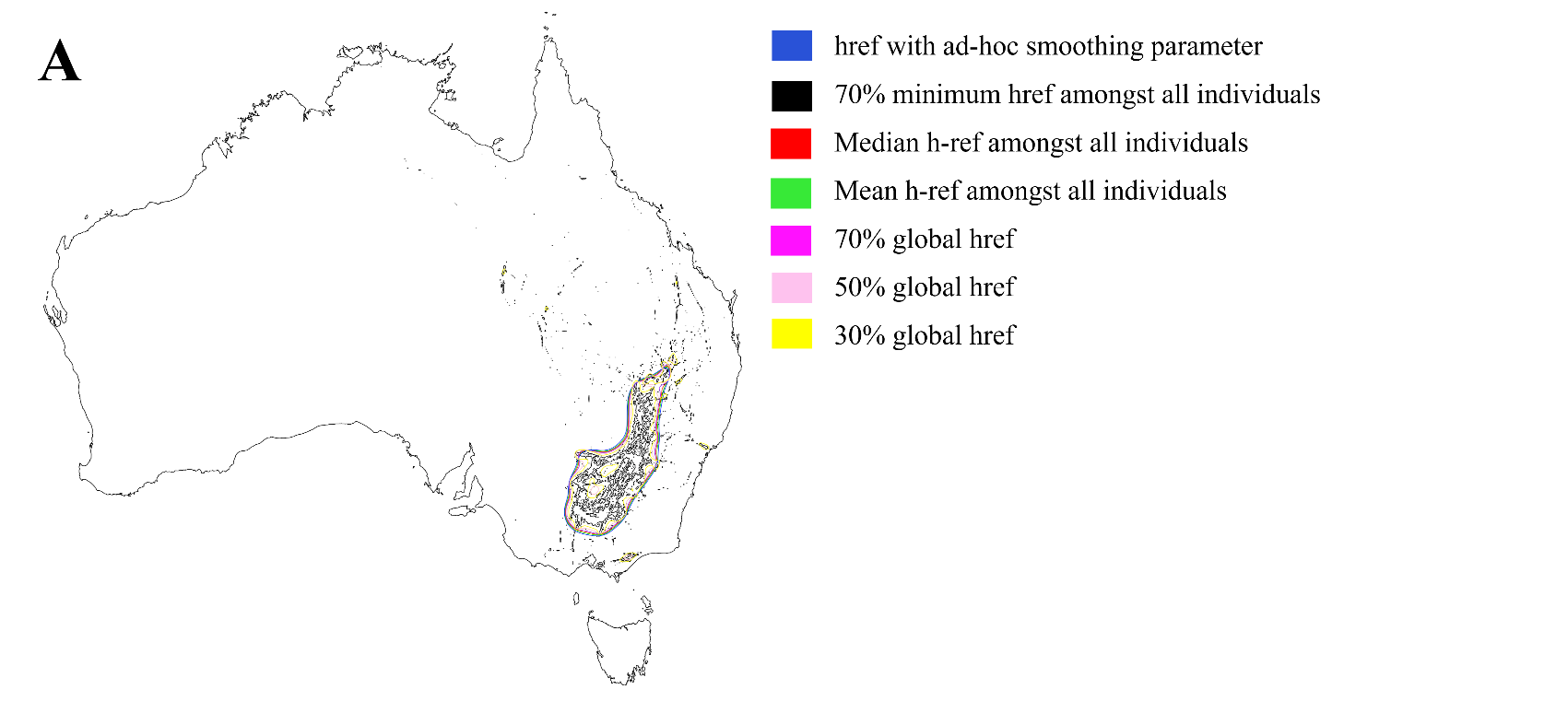

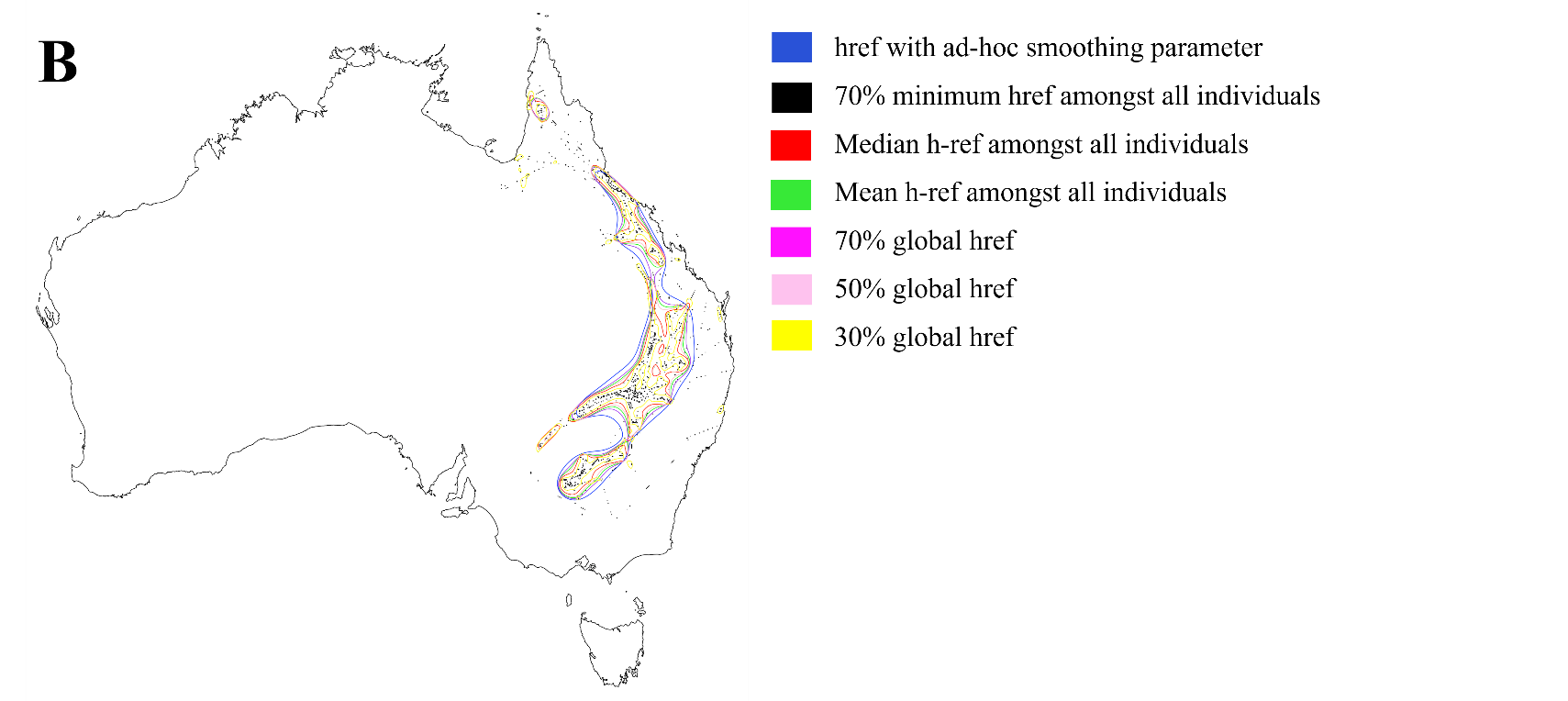


Figure S2.2 KED50s generated using different href values (legend denotes href method used to generate each KED50). A = straw-necked ibis, B = royal spoonbill.


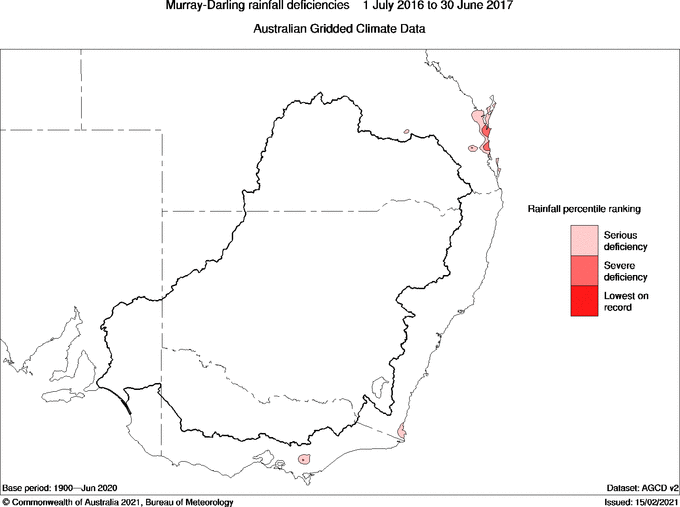


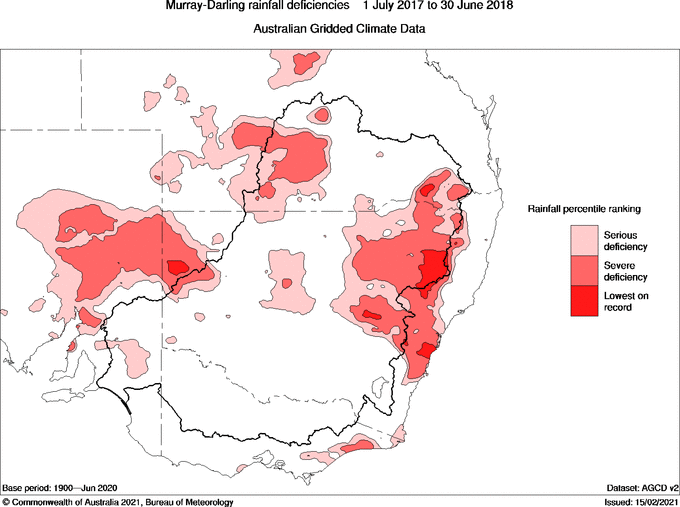


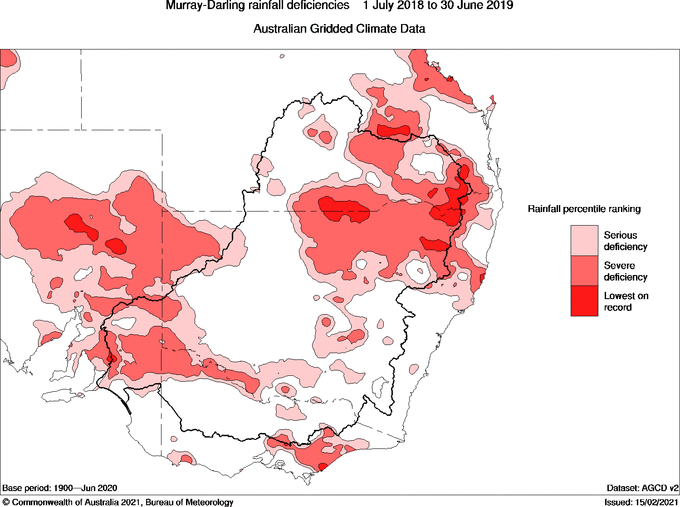


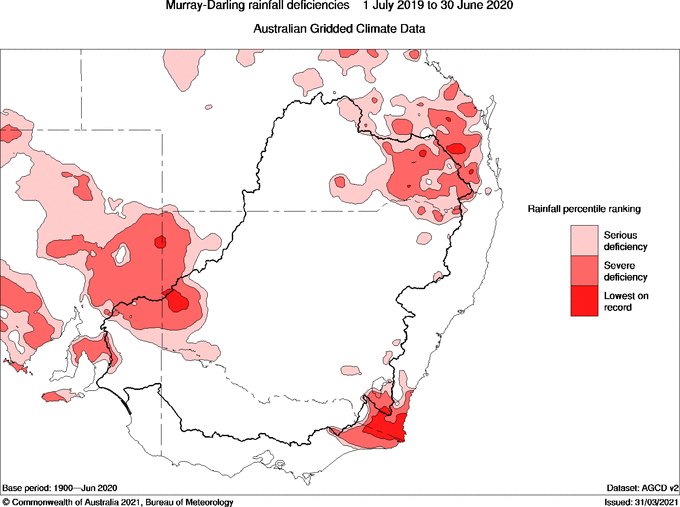


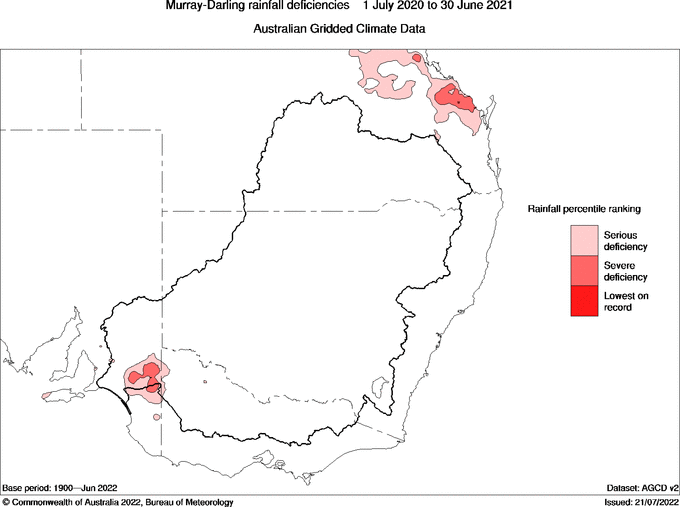


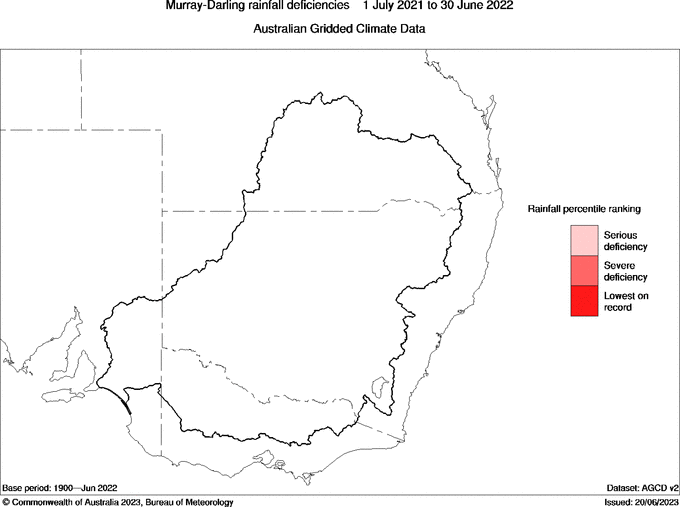


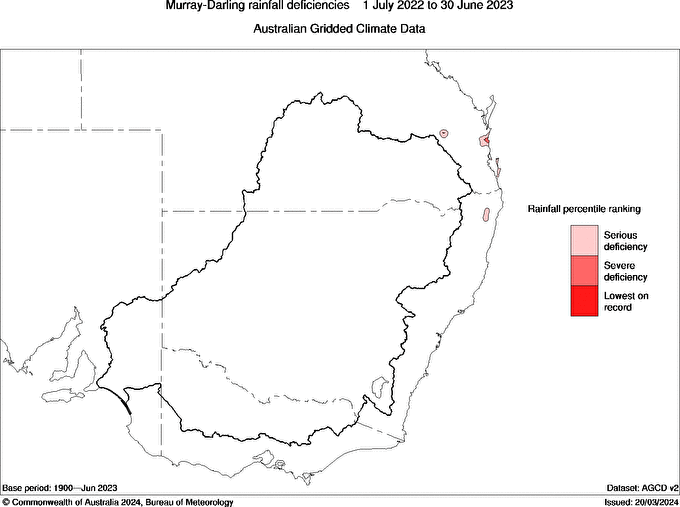


Figure S2.3 Maps showing annual drought patterns in the Murray Darling Basin (MDB) divided into 12-month periods from 1 July to 30 June for the years that waterbirds were tracked. Based on the above we classed 2017-2018, 2018-2019 and 2019-2020 as dry years (substantive areas of the MDB included in a drought category) and 2016-2017, 2020-2021, 2021-2022 and 2022-2023 as wet years (little to none of the MBD included in a drought category). Map source: Australian Government Bureau of Meteorology <http://www.bom.gov.au/climate/maps/rainfall> accessed 9 April, 2024.


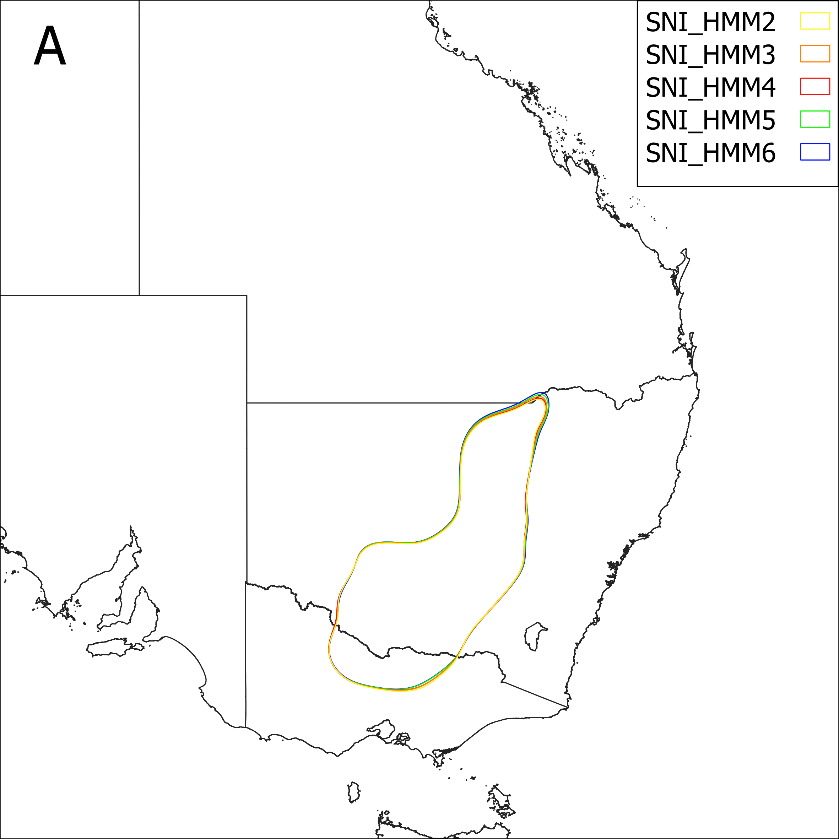

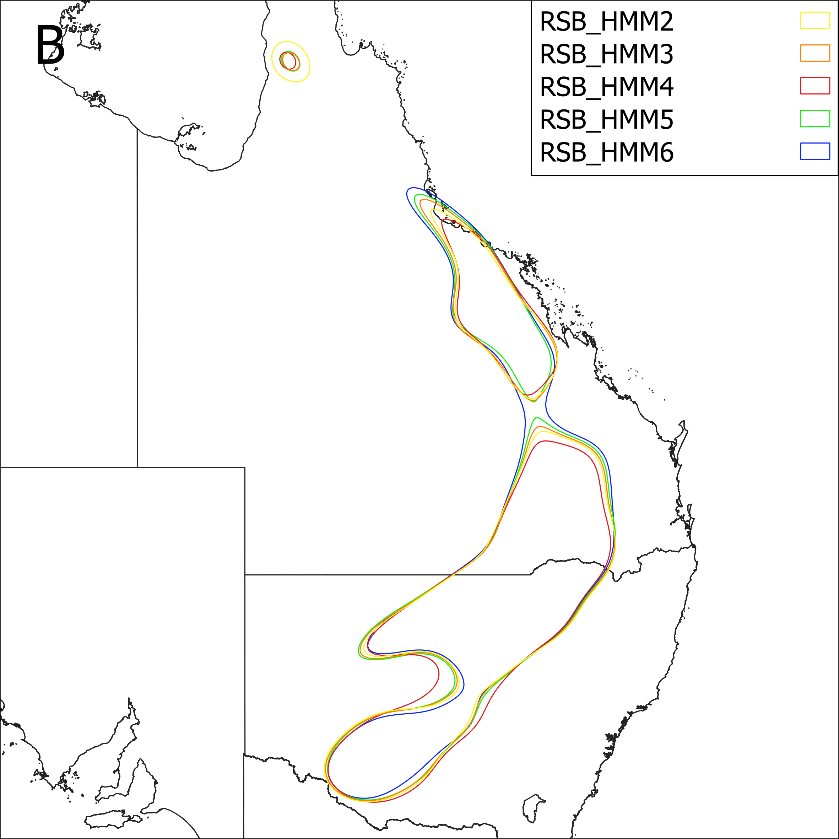


Figure S2.4 Comparison of KED50 for Hidden Markov Models (HMMs) with 2, 3, 4, 5 and 6 states for straw-necked ibis (SNI; A; Table S1.4) and royal spoonbill (RSB; B; Table S1.4).


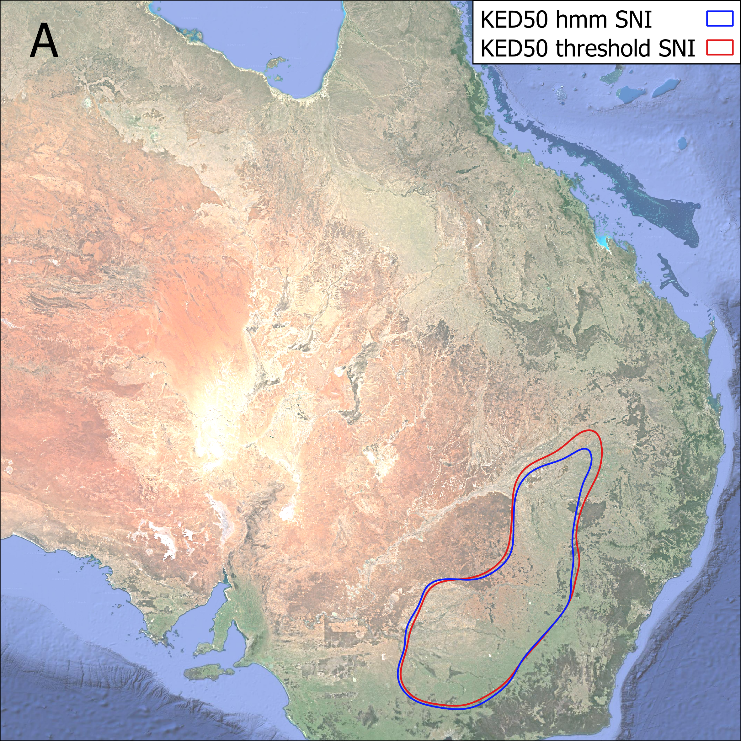

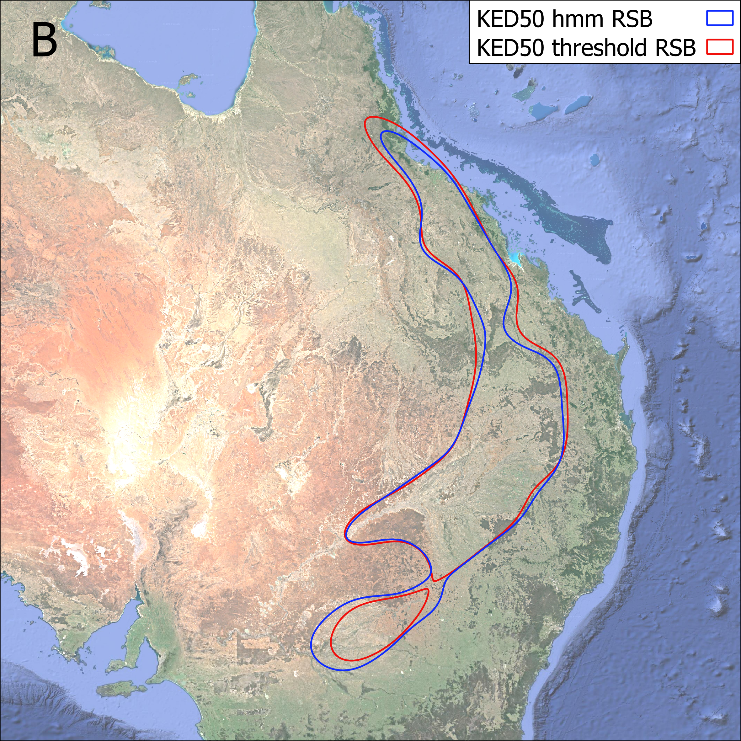


Figure S2.5 50% contour of the kernel density estimate (KED50) for SNI (A) and RSB (B) generated using a dataset of long-distance movements subset from a dataset of lines of the maximum distance that a bird had travelled in the 24 hours from midnight to midnight (SNI) or midday to midday (RSB) based on: i) fourth movement class from a five-state Hidden Markov Model (SNI; Table S1.4; blue) and sixth movement class from a six-state Hidden Markov Model (RSB; Table S1.4; blue) and ii) a thresholding approach that defined long-distance movements defined as the median movement length amongst all lines plus two standard deviations (Table S1.2; red).


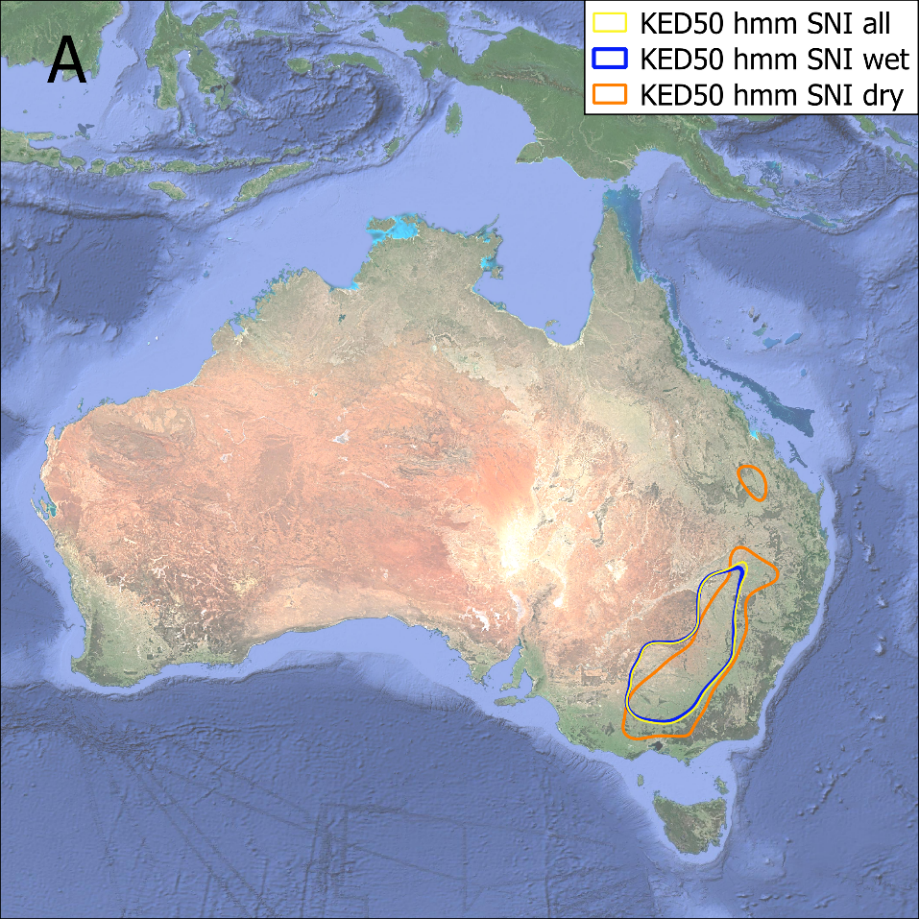

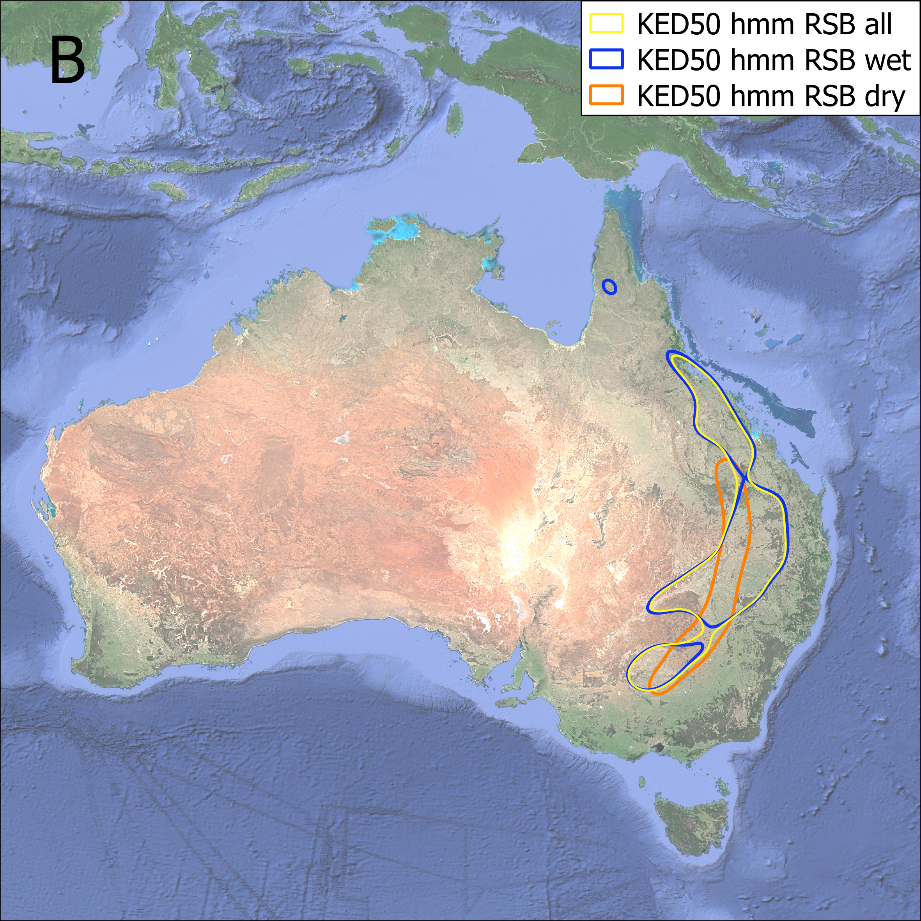


Figure S2.6 KED50 for SNI in all (yellow), wet (blue) and dry (orange) years (A). KED50 for RSB in all (yellow), wet (blue) and dry (orange) years (B). Wet years = 2016-2017, 2020-2021, 2021-2022 and 2022-2023. Dry years = 2017-2018, 2018-2019 and 2019-2020. ‘Years’ defined as 1 July to 30 June.


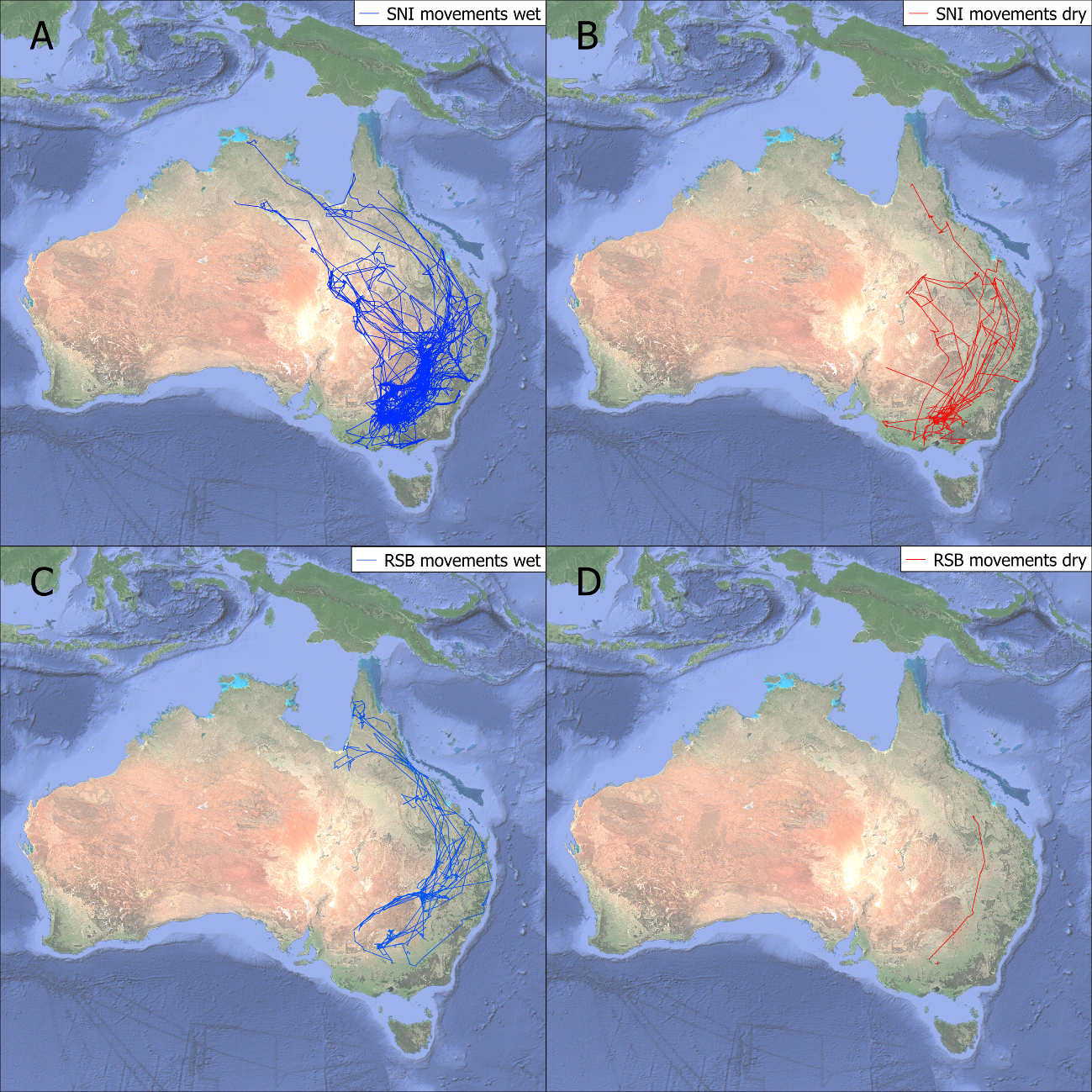


Figure S2.7 Long-distance movements of SNI in wet (A) and dry (B) years. Long-distance movements of RSB in wet (C) and dry (D) years. Wet years = 2016-2017, 2020-2021, 2021-2022 and 2022-2023. Dry years = 2017-2018, 2018-2019 and 2019-2020. ‘Years’ defined as 1 July to 30 June.


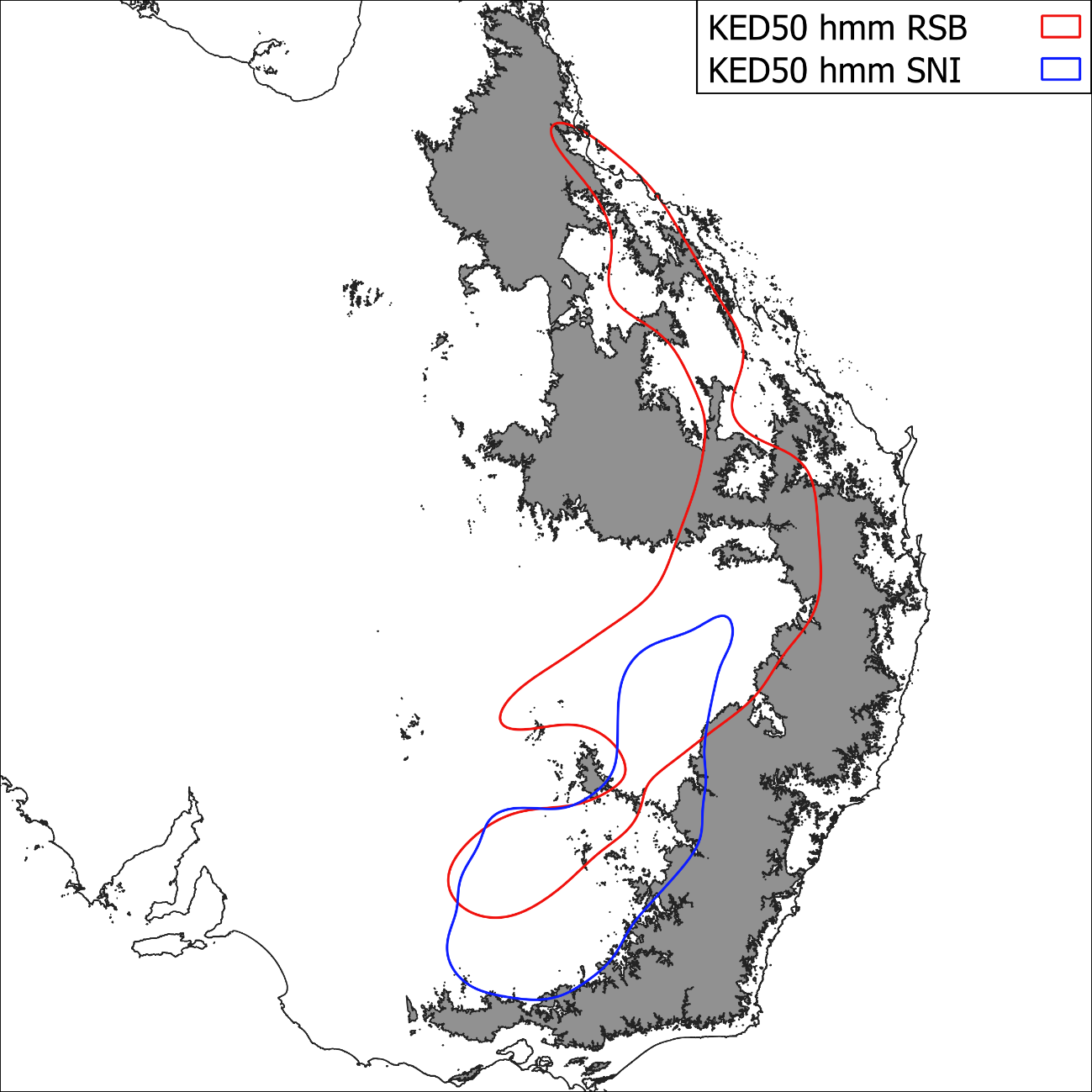


Figure S2.8 Australia’s Great Dividing Range mapped here (grey) as the 300 m elevation contour (derived from BRS, 2009). KED50 for SNI (blue) and RSB (red) are also shown.
